# Genetics and Epigenetics of Atopic Dermatitis: An Updated Systematic Review

**DOI:** 10.3390/genes11040442

**Published:** 2020-04-18

**Authors:** Maria J Martin, Miguel Estravís, Asunción García-Sánchez, Ignacio Dávila, María Isidoro-García, Catalina Sanz

**Affiliations:** 1 Institute for Biomedical Research of Salamanca (IBSAL), 37007 Salamanca, Spain; mjmartinma@saludcastillayleon.es (M.J.M.); estravis@usal.es (M.E.); idg@usal.es (I.D.); catsof@usal.es (C.S.); 2 Network for Cooperative Research in Health–RETICS ARADyAL, 37007 Salamanca, Spain; 3 Department of Biomedical and Diagnostics Sciences, University of Salamanca, 37007 Salamanca, Spain; 4 Department of Immunoallergy, Salamanca University Hospital, 37007 Salamanca, Spain; 5 Department of Clinical Biochemistry, University Hospital of Salamanca, 37007 Salamanca, Spain; 6 Department of Medicine, University of Salamanca, 37007 Salamanca, Spain; 7 Department of Microbiology and Genetics, University of Salamanca, 37007 Salamanca, Spain

**Keywords:** atopic dermatitis, genetics, epigenetics, skin barrier, genetic association studies, DNA methylation, omics

## Abstract

Background: Atopic dermatitis is a common inflammatory skin disorder that affects up to 15–20% of the population and is characterized by recurrent eczematous lesions with intense itching. As a heterogeneous disease, multiple factors have been suggested to explain the nature of atopic dermatitis (AD), and its high prevalence makes it necessary to periodically compile and update the new information available. In this systematic review, the focus is set at the genetic and epigenetic studies carried out in the last years. Methods: A systematic literature review was conducted in three scientific publication databases (PubMed, Cochrane Library, and Scopus). The search was restricted to publications indexed from July 2016 to December 2019, and keywords related to atopic dermatitis genetics and epigenetics were used. Results: A total of 73 original papers met the inclusion criteria established, including 9 epigenetic studies. A total of 62 genes and 5 intergenic regions were described as associated with AD. Conclusion: *Filaggrin* (*FLG*) polymorphisms are confirmed as key genetic determinants for AD development, but also epigenetic regulation and other genes with functions mainly related to the immune system and extracellular matrix, reinforcing the notion of skin homeostasis breakage in AD.

## 1. Introduction

Atopic dermatitis (AD), also known as atopic eczema, is a common inflammatory skin disorder that affects up to 15–20% of children [1] and 7–10% of adults [2] in developed countries. AD typically develops during childhood and is characterized by recurrent eczematous lesions with intense itching [1]. It is considered the first step of the atopic march, associated with an increased risk of developing allergic rhinoconjunctivitis, asthma, or food allergy [3]. Worldwide, the prevalence of AD used to be higher in countries with higher incomes. However, due to the globalization process and a more westernized way of life, an increase of AD prevalence in low-income countries of Africa and East Asia has been reported, stressing the role of environment together with genetic and immunologic factors in the pathogenicity of AD [4].

AD is a heterogeneous disease. Thus, interactions between susceptibility genes, environmental factors, impaired barrier skin integrity, skin microbiota, and immune deregulation have been proposed to explain the nature of AD [5]. An urban way of life is one of the most clearly related environmental factors, as supported by a consistently higher incidence of eczema in urban versus rural areas [6]. Diet is also a risk factor, and a regular intake of fresh fruit and fish during pregnancy and childhood has shown some effectiveness in preventing AD [7,8,9]. Furthermore, it has been reported that a family history of asthma, hay fever, or eczema is associated with AD in the offspring, the risk being higher when both parents suffer from eczema [10].

AD is associated with atopic comorbidities such as asthma, allergic rhinoconjunctivitis, and food allergy, as well as with non-atopic entities such as inflammatory diseases and psychological disorders [11,12]. Genetic association studies have confirmed that atopic comorbidities share genetic susceptibility [13,14]. The heritability of AD in twin studies was estimated to be nearly 75%, and the association between asthma and AD was nearly 85% explained by genetic pleiotropy [15].

Two main reviews about the genetics of AD have been performed in the last decade. The first one was published in 2010 and compiled all the existent genetic studies related to AD [16], reporting variants in 81 genes, 46 of which had shown positive association with the disease. In 2016, Bin and Leung published an update on the topic, including genetic and epigenetic studies from 2009 to June 2016 [17]. The aim of this systematic review is to compile the most recent publications on the genetics of atopic dermatitis, also including epigenetic studies. Original articles about genetic variation and polymorphisms in patients with atopic dermatitis or atopic eczema were sought. Both adult and children studies were included. Comparison to healthy control was preferred, but not all studies performed it. Although literature reviews were excluded, we analyzed some meta-analyses due to the valuable information they contained.

## 2. Materials and Methods

This systematic review has been performed using the PRISMA guidelines for Systematic Reviews and Meta-Analysis 2009 checklist and GRADE recommendations [18]. 

Original articles and meta-analyses indexed from July 2016 to December 2019, describing genetic or epigenetic aspects of atopic dermatitis, were searched. We identified eligible studies using the following inclusion criteria: (1) primary study or meta-analysis, (2) written in English or Spanish, (3) human subjects, both children and adults, (4) patients suffering from atopic dermatitis or atopic eczema, and (5) studies describing mutations, single nucleotide polymorphisms (SNPs), or epigenetic modifications in association with disease onset, severity, or prevalence in the population. The exclusion criteria were: (1) animal, in vitro or in silico studies, (2) review articles, (3) proteomics or expression analysis without epigenetic/genotyping study, (4) articles focused in other diseases, such as psoriasis or ichthyosis vulgaris, in which AD was merely mentioned, and (5) articles whose full-text version was not available to us.

We performed the literature search between December 2019 and January 2020 in PubMed, Cochrane Library, and Scopus databases, using the following terms: “atopic dermatitis” OR “atopic eczema” AND “gene” OR “genetic” OR “mutation” OR “epigenetic” OR “DNA methylation” OR “sequencing” OR “microRNA” OR “polymorphism” OR “genome-wide association study” OR “microarray” OR “gene profiling”.

Three authors individually reviewed the database search results, assessing titles, evaluating abstracts, and considering or not the study for full review. Any disagreements in either the title/abstract or the full manuscript review phases were resolved by consensus. All eligible studies were formally evaluated and included in this systematic review.

The authors independently evaluated the quality appraisal and graded the risk of bias of the included studies. The risk of bias was assessed by Rob2, the recommended tool to assess the risk of bias in randomized trials included in Cochrane Reviews [19], slightly adapted by the authors to fit the nature of the selected articles. Studies were classified as low, moderate, or high risk of bias.

Quality was assessed using the Newcastle–Ottawa scale [20]. Each study was awarded one point per positive item, according to the scale. Scores over 6 merited “high quality”; those below 4 were considered as “low quality”; the rest being classified as “moderate”.

Epigenetic methodology has some peculiarities that prevent the application of bias and quality scoring by the commonly used scales. Some notes regarding this will be mention when discussing the selected epigenetic studies.

Gene pathway analysis of the found genes was performed using FunRich [21], Reactome [22], and STRING [23].

## 3. Results

### 3.1. Selection, Bias and Quality of Articles

**Figure 1:**
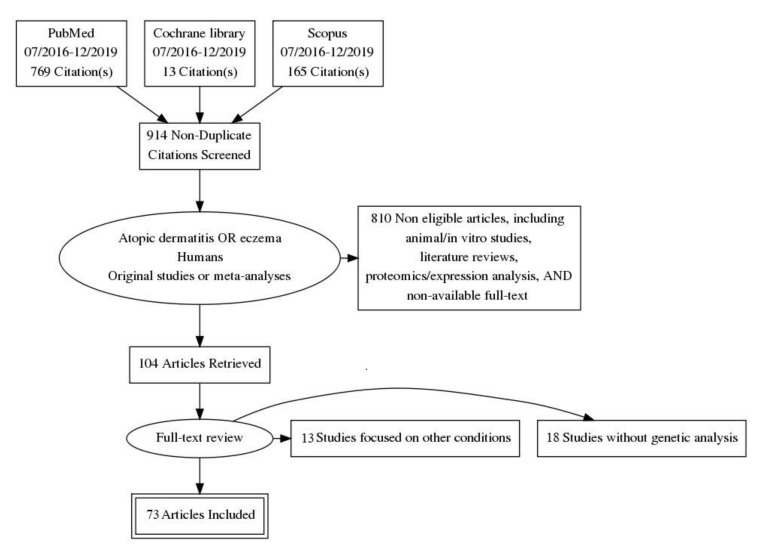
The flow diagram depicts the flow of information through the different phases of the systematic review. It maps out the number of records identified, included and excluded, and the reasons for exclusions.

The database search yielded 914 articles (Figure 1). Atopic eczema was used for the search engine as a synonymous term. After title and abstract review, 810 articles were rejected since they did not fulfill eligibility criteria, i.e., those describing animal or in vitro studies, literature reviews, analysis of protein or gene expression, and articles written in languages other than English or Spanish. Therefore, 104 articles qualified for full text review. Of those, we eliminated 13 studies that mentioned AD in comparison to other conditions but were not fully dedicated to it and 18 studies that did not perform any genetic association with the disease. As a result, 73 articles were evaluated. Out of 73 studies, 11 were related to epigenetics [13,24,25,26,27,28,29,30,31,32,33], 39 were candidate gene studies [34,35,36,37,38,39,40,41,42,43,44,45,46,47,48,49,50,51,52,53,54,55,56,57,58,59,60,61,62,63,64,65,66,67,68,69,70,71,72,73], 5 were genome-wide association studies (GWAS) [5,13,74,75,76], whole-exome sequencing (WES) was performed in 7 articles [77,78,79,80,81,82,83], and phenome-wide association sequencing was done in 1 article [84]. Four studies described results from next-generation sequencing (NGS) [85,86,87,88], and 2 showed analyses of copy number variations (CNV) [89,90]. Besides, 6 meta-analyses were also included [91,92,93,94,95,96].

A description of the 64 selected non-epigenetic studies is presented in Table 1. Epigenetic articles are summarized in Table 2.

As mentioned above, we followed the Cochrane guidelines to assess the risk of bias of the selected non-epigenetic studies, using the current version of the Rob2 tool [19]. As this tool has been developed for randomized trials, the authors decided to make some assumptions in order to adapt it to the specific nature of the genetic analysis. Taking into account that our main concern with respect to bias referred to the lack of appropriate controls or non-adequate genetic or epigenetic techniques for achieving the intended aim, we responded to questions about intervention or randomization consequently. Therefore, a study was classified as high risk when healthy controls were missing or the methodology to analyze the samples was not clearly explained in the text.

**Figure 2:**
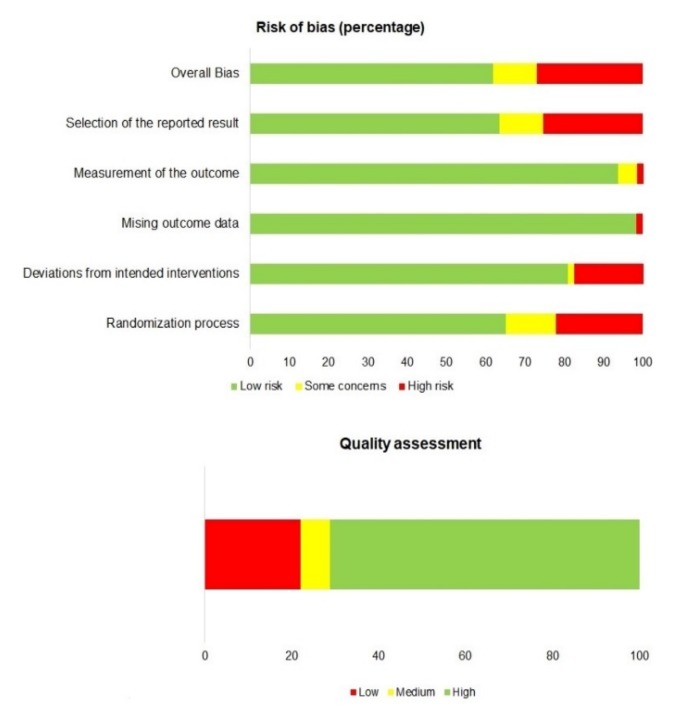
Risk of bias (upper panel) and quality assessment (bottom panel) of the selected genetic studies, as a percentage of the total.

Under these conditions, 20.3% of the non-epigenetic studies were considered at a high risk of bias. Healthy controls were not included in 14 studies, some of them referring to very few patients. One of the studies had no reference to the sample size, and another one did not describe the used methodology. We found some concerns in 4 studies, mainly referring to the selection of subjects. The rest of the selected studies (71.9%) accomplished our criteria for low risk of bias (Figure 2, Table S1).

Correspondingly, 71.2% of the articles merited high quality after running the NOS questionnaire (Table S2). Overall, the representativeness of the cases was the better-scored category. Thirteen articles were considered low-quality studies, mainly due to failed selection and definition of relevant controls. 

### 3.2. Genetic Studies

A total of 62 genes and 5 intergenic regions were described as associated with AD in the selected studies; 32 of them were related to the disease for the first time during this period. Among them, filaggrin was the most widely reported gene, being over 90% of the other genes cited in only one article. 

**Figure 3:**
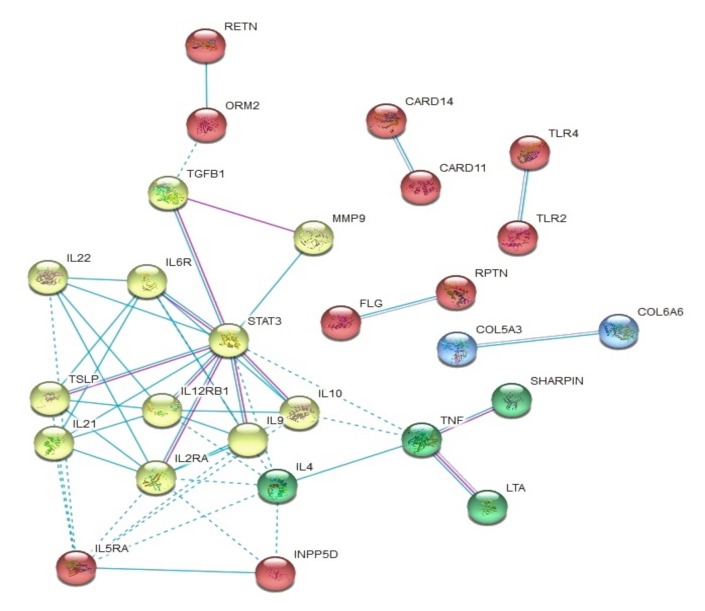
Functional association protein networks in STRING software (STRING consortium: Swiss Institute of Bioinformatics, Lausanne, Switzerland; Novo Nordisk Foundation CPR, Copenhagen, Denmark; EMBL Heidelberg, Germany) established from the genes reported in the selected studies. The diverse clusters are colored differently. Protein–protein interactions are drawn in blue, when obtained from curated databases, or purple if experimentally determined. Inter-cluster edges are represented by dashed-lines.

The interaction analysis performed by STRING showed some connectivity enrichment of the listed proteins (*p*-value < 1e-16). The network was clustered using the k-means method. Clustering results are shown in Figure 3 (non-linked proteins were removed from the graph). Three main clusters were found. The most populated included cytokines related to STAT3, with connections with ORM2 and RETN, and TNF.

**Figure 4:**
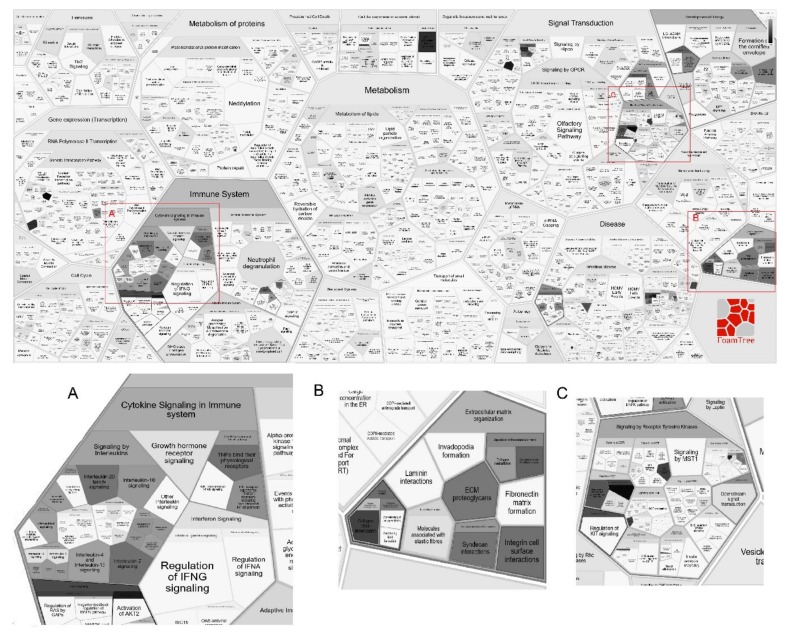
Reacfoam shows a high-level pathway overview of the genes reported in the selected studies. Significantly enriched pathways are shown in dark shade. The three main pathways have been zoomed in as (**A**) cytokine signaling in immune system, (**B**) extracellular matrix organization, and (**C**) signaling by receptor tyrosine kinases.

With respect to the pathway analysis, a genome-wide overview from Reactome is shown as a Reacfoam in Figure 4 (for a higher resolution, a zoomable pdf version is available as Figure S1). Reacfoam shows a high-level pathway overview visualization based on Voronoi tessellation. Darker functions correspond to those that are over-represented in the list of genes identified in the selected studies, i.e., immune system, developmental biology, signal transduction, and extracellular matrix (ECM). Immune system functions stood up among the high hierarchy pathways (False Discovery Rate (FDR) 2.21e-6; *p*-value 2.22e-8). 

The most significant pathways were related to signaling by interleukins (FDR 1.7e-11; *p*-value 4.17e-11) or different variants, i.e. IL-4/IL-13 (FDR 7.02e-10; *p*-value 5.2e-10), IL-2 (FDR 3.89e-5; *p*-value 4.8e-7) or IL-12 signaling (FDR 1.42e-4; *p*-value 2.11e-6). Identifiers found in the former pathway were *IL6R, IL10, TGFB1, TNF, STAT3, ADAM33, IL4, IL13*, and *MMP9*. *IL5RA, STAT3, IL2RA, INPP5D, IL9,* and *IL21* were associated with the IL-2 signaling pathway, while *MIF, STAT3, IL10*, and *IL12RB1* were the IL-12 pathway entities found in the analysis. *IL22* and *INNP5D* had not been related to AD before 2016. Moreover, enrichment was also found in transcriptional regulation of granulopoiesis (FDR 0.074; *p*-value 0.009), the process leading to the production of neutrophils, eosinophils, and basophils. Signaling cascades by *MAPK1* (FDR 0.04; *p*-value 0.002) and *FGFR* (FDR 0.04; *p*-value 0.003) were also significantly enriched. The related entities found in the analysis were *STAT3, FLG, IL2RA, IL5RA, IL6R,* and *MCM10*.

Seven out of the 62 genes described in the period covered by this review have been curated with functions in pathways related to extracellular matrix organization (FDR 4.58e-2; *p*-value 3.81e-3). Interestingly, 6 of these genes (*COL5A3, COL6A6, KDR, MCM10, MMP9,* and *STS*) had not been associated with AD yet, and only TGF-β1, involved in a broad spectrum of pathways, had been previously associated [97].

**Figure 5:**
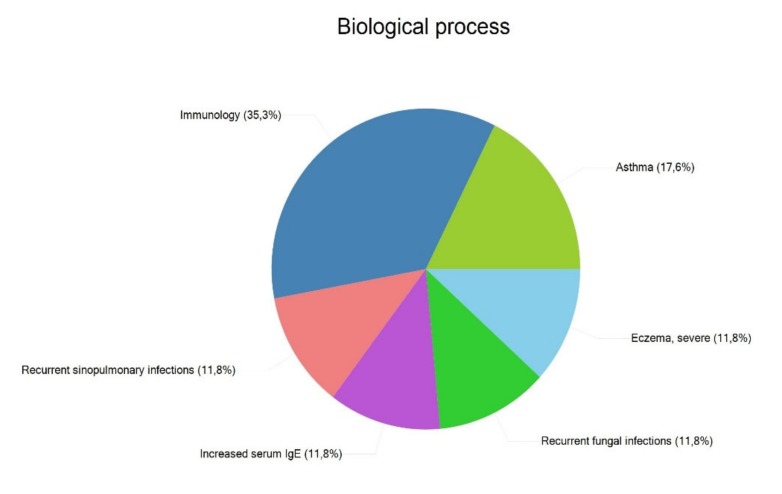
Most relevant biological processes involving the reported genes. Percentages indicate the number of genes with respect to total that is included in each process.

We also performed an analysis of disease-related genes using the FunRich software. The results are shown in Figure 5. The appearance of DOCK8 in all these biological processes stands out, taking into account that it has been described as related to AD only in one study reporting a single case [77]. COSMIC analysis located 75.4% of genes in the skin (*ACTL9, ADAM33, ADCY10, C11orf30, CARD11, CARD14, CLDN1, COL5A3, CRNN, CUX2, CYP27A1, DEFB1, DOCK8, FLG, GSDMB, IL12RB1, IL22, IL2RA, IL4, IL5RA, IL6R, IL9, KDR, LILRA6, LRRC32, MAST2, MCM10, MMP9, MTF1, NLRP2, ORM2, PANX3, PHLDB1, PRR5L, RPTN, RTEL1, SPINK5, STS, TCHHL1, TGFB1, TLR2, TLR4,* and *TNF*). 

Additionally, 17 of the new AD identified genes could not be ascribed to significant biological functions, i.e., *CARD14, CRNN, TCHHL1, RPTN, PANX3, PHLDB1, LILRA6, NLRP2, MTF1, LTA, MAST2, DOCK8, CUX2, ADCY10, VSTM1,* and *RTEL1.*

### 3.3. Filaggrin

During the period of this revision, we have identified 33 studies on the *filaggrin* (*FLG)* gene association with AD. It is remarkable that 16 novel mutations have been reported [56,81,85,88].

#### 3.3.1. *Filaggrin* Mutations and Other Allergic Diseases

Eleven studies analyzed the association between *FLG* mutations and allergic sensitization, showing that *FLG* alleles conferred an increased risk, mainly in children with eczema [13,39,41,44,47,50,51,58,70,72,76]. In Polish children, Debinska et al. showed that several *FLG* mutations predisposed patients to eczema plus asthma, increasing more than 6-fold the risk of this complex phenotype (*p*-value 0.043) [72]. By contrast, such increased risk of asthma in *FLG* mutation was not confirmed in adult twins, although the risk of having AD was increased in those individuals with asthma, compared to individuals without asthma (27.6% vs. 18.5%; OR 1.68, 95% CI 1.12–2.52; *p*-value 0.012) [58]. Chan et al. showed a significant effect of *FLG* loss-of-function (LoF) mutations on both asthma and rhinitis at ages 1, 2, 4, 10. and 18 years, particularly at the age of 10 years (RR 1.96; 95% CI 1.70–2.26; *p*-value 0.003), early eczema being a requisite to suffer asthma at all ages [70]. 

Ferreira et al. carried out GWAS on individuals suffering from asthma, hay fever, and eczema to identify shared risk variants. The rs6181676[A] *FLG* variant was 1.32-fold more common in individuals suffering only from eczema when compared to individuals suffering only from hay fever (*p*-value 7.2e-8), and 1.26-fold comparing with asthma-only cases [13]. Two *FLG* single nucleotide polymorphisms (SNP), rs71626704 and rs76413899, were significantly associated with a history of asthma and cheilitis (*p*-value 0.002 and *p*-value 0.003, respectively) and rs62623409 and rs71625199 SNPs were associated with sensitization to environmental allergens (*p*-value 0.038 and *p*-value 0.008, respectively). Rs11584340 was associated with an increase of eosinophil-derived neurotoxin serum levels in allergic rhinitis patients and eosinophilic cationic protein serum levels in asthmatic patients [41]. Park et al. reported an association between *FLG* LoF mutation and early onset of asthma and AD [87]. The association between an *FLG* mutation and IgE sensitization to peanut at age 4 years (OD, 1.88; 95% CI 103–3.44), but not to other allergens was reported by Johansson et al. [39]. Equally *FLG* mutations were significantly associated with elevated IgE in a population of Korean patients with AD (>200KIU/L and/or MAST-CLA>+, *p*-value 0.005), palmar hyperlinearity (*p* < 0.001), and a family history of allergic disease (*p*-value 0.021) [50]. However, there was no significant difference in IgE levels between AD patients with non-mutated *FLG* and those carrying *FLG* LoF mutations (*p*-value 0.062) [44]. 

#### 3.3.2. *Filaggrin* Mutations and Early Onset of AD

Patients who carried *FLG* mutation alleles are associated with early-onset AD [62,72]. Wan et al. found a dose-dependent association between the number of common *FLG* mutations and early onset [62]. In a Finish population, the combination of *FLG* mutations was shown to be significantly associated with early-onset of AD (<2 years) (OR 4.15, *p*-value 1.82e-10) and asthma (OR 2.76, *p*-value 1,57e-6) [47]. In two independent cohorts, *FLG* LoF mutations were associated with subphenotypes of AD. Thus, in a cohort study of 14,701 children from Avon (UK), the strongest association was detected with early-onset-persistent AD (OR 4.31; 95% CI 3.29–5.63; *p*-value 2e-26) and in a Dutch cohort of 3963 children, only the group of children with early-onset-late-resolving AD was associated with *FLG* LoF mutations (OR 5.63; 95% CI 2.65–11.95; *p*-value 7e-6) [76]. 

Additionally, it has been demonstrated that *FLG* expression in umbilical cord blood was associated with eczema development in infancy, being significantly lower in children with *FLG* variants when compared to children with wild-type *FLG* genotype (*p*-value 0.007) [75].

#### 3.3.3. *Filaggrin* Mutations and other Skin Diseases

Andersen et al. studied the prevalence of *FLG* null mutations in adult patients with actinic keratosis (AK), premalignant intra-epidermal skin lesions that can progress into squamous cell carcinomas (SCCs). In their study, 7.5% AK patients had an *FLG* LoF mutation, of whom only the homozygous mutation carriers (0.8%), but not heterozygous, showed an increased risk of AK compared with wild-types (*p*-value 0.0017) [65]. Elhaji et al. found a significant association between the R501X mutation with polysensitivity in contact dermatitis when three or more positive patch test reaction occurred (8.5% patients vs. 4% controls; *p*-value 0.008) [91]. 

*FLG* mutations were significantly associated with palmar hyperlinearity in a population of Korean patients with AD (*p* < 0.001) [50], and also in a Finish population (OR 4.67, *p*-value 1.46e-5) [47].

The specific variant rs558269137 was exclusively detected in Italian children with AD and *Molluscum contagiosum* virus (MCV) infection, while rs374910442, rs138055273, rs113136594, and rs11584340 variants were found both in AD children and AD plus MCV-infected children [48]. 

#### 3.3.4. *Filaggrin* Mutations and Eczema Severity

Seven studies analyzed the association between *FLG* mutations and eczema severity [48,50,53,71,87,88,90]. Chang et al. found that *FLG* LoF homozygotes and heterozygotes were less likely to report periods of skin clearance (OR 0.20; 95% CI 0.07–0.55) and more likely to report frequent steroid use (OR 3.18; 95% CI 1.22–8.30) [71]. 

Specific gene variants of *FLG* have been associated with moderate to severe SCORAD (Scoring Atopic Dermatitis) indexes. Rs145627745, rs79808464, rs150957860, and rs145828067 variants were entirely associated with moderate disease severity (SCORAD 25-50), rs747005144 variant was associated with severe disease (SCORAD >50), whereas rs374910442, rs138055273, rs183942200, rs1584340, and rs113136594 variants were associated with both moderate and severe disease [48].

In a population of African American (AA) children with AD and *FLG* LoF, 77% exhibited severe AD (SCORAD >50) [90]. A negative effect on the success of the immunosuppressive treatment was reported in *FLG* mutated patients when compared with those without *FLG* mutations [53]. When exploring the correlation between Eczema Area and Severity Index (EASI) and *FLG*-related AD in Korea, contradictory results were reported [50,87]. In addition, Wong et al. did not find any significant association between *FLG* LoF and the severity of AD [88].

#### 3.3.5. *Filaggrin* Mutations and Ethnicity Risk Factors

Fifteen studies were carried out on European ancestor populations [12,13,36,39,44,47,48,53,58,70,72,75,76,91,98], 11 studies in Asia populations [37,45,50,51,54,56,64,67,81,87,88], 2 studies in African ancestor populations [85,90], and 3 studies in mixed populations [62,71,73]. The most prevalent LoF in patients with European ancestors were R501X, 2282del4, S3247X and R2447X, analyzed in 27 studies [12,13,36,37,39,44,47,48,50,53,54,56,58,60,62,64,70,71,72,73,75,76,81,85,88,90,91]. Other ethnic ancestors and rare variants were analyzed in 15 studies [37,41,45,47,50,51,53,54,56,64,81,85,87,88,90].

Elbert et al. carried out a study to analyze the association of ethnic origin with *FLG* mutations and environmental risk factors in children from multiethnic origins but living in the Netherlands, showing that minority ethnicity children had a higher risk of eczema than Dutch children [73]. Gimalova et al. studied LoF variants in Russians and Tartars AD patients, reporting that c.2282del4 was the most prevalent mutation in both populations, whereas R501X and R2447X mutations were rare [36]. In India, a study of the association between *FLG* mutations and hand eczema showed that mutations in S2889X constituted 96.4% of all *FLG* mutations, while European mutations were not found [37]. 

An overview of the genetic map and geographic distribution of *FLG* mutations across East Asia found that 3321delA is a pan-Asian mutation [45]. K4022X, the most prevalent *FLG* mutation in Korea and northern China, showed a south-to-north distribution gradient. In contrast, c.6950del8 showed the reverse effect. On the other hand, S2554X, S2889x, S3296X, and Q1701X mutations were Japanese-specific. These *FLG* mutations were associated with an increased risk of AD but did not confer a risk of asthma [45]. 

On et al. carried on a study of *FLG* mutations previously detected in Korean, Japanese, and Chinese patients on seventy Korean patients with AD. Four LoF mutations (3321delA, K4022X, S3296X, and S2889X) were identified in 15.7% patients [50]. The most commonly detected variants in Korean patients with AD were 3321delA (9.1%) and Y1767X (1.6%), K4022X (4.3–4.5%) [50,51,87]. Interestingly, Y1767X was only found in AD patients, whereas K4022X was found in both patients and controls [51]. 

Pigors et al. analyzed the genetic scheme of AD patients from the South Asian Bangladeshi community using WES combined with rare variant enrichment analysis [81], showing that *FLG* carried the highest number of enriched dominant (OR 12.1; *p*-value <0.0001) and recessive (OR 43.4; *p*-value <0.0001) LoF mutations. Three of the LoF mutations were previously unreported (S923Ffs*2, T1545Qfs*163, and S2352X). Furthermore, these genetic data revealed intrafamilial heterogeneity with multiple *FLG* variants often segregating within the Bangladeshi families with AD [81]. 

The common European *FLG* LoF R501X and 2282del4 were significantly associated with the risk of developing AD (OR 11.29, *p*-value 0.00022 and OR 2.66, *p*-value 0.00016, respectively) in Finnish patients [47]. In addition, having two 12-repeat alleles (rs12730241) was found to be significantly associated with a higher risk of AD (OR 1.96, 95% CI 1.36–2.81, *p*-value 0.00056) [47]. 

Using massively parallel sequencing, Margolis et al. identified nine *FLG* LoF variants in AA children, including 6 newly reported and 3 previously described, suggesting multiple and rare *FLG* LoF variants. Those children with *FLG* LoF variants had more persistent AD than wild-type children for *FLG* LoF [85]. These findings were supported by Mathyer et al., who identified five *FLG* LoF variants in 9 heterozygous AA AD patients (488delG, R501X, R826X, S3101X, and S3316X) [90]. 

New mutations were also found in Japanese (R826X) and Korean (S2889X) ichthyosis vulgaris (IV) patients [56]. Although the R826X mutation has not been detected in Japanese and Korean AD patients to date, it had been previously reported in Chinese and AA populations [95,99], suggesting that it is not a population-specific *FLG* mutation. Japanese and Korean patients shared 4 *FLG* mutations, Gly1109Glufs*13, Ser2889X, Ser3296X, and Lys4022X, being the latter more frequent in Korean than in Japanese AD or IV patients [56].

A robust and cost-effective high-throughput PCR-based method using microfluidics technology and NGS was applied to study the *FLG* coding region in cohorts of Chinese, Malaysian, and Indian AD patients living in Singapore. Thirty-three *FLG* LoF variants were identified in Chinese subjects, being 5 of them novel mutations. Unreported *FLG* LoF variants in Indian and Malaysian patients confirmed the diversity depending on the ethnic group [88].

### 3.4. Other Genes

Significant associations with AD have been reported for most of the analyzed genes and variants. Thus, *ACTL9, C11orf30, IL6R, IL21, IL22, INPP5D, KIF3A, OVOL1, PRR5L, PPP2R3D*, and *STAT3* were investigated in two cohorts, ALSPAC and PIAMA [76]. *ADCY10, CUX2, MAST2, MCM10, MTF1, ORM2, PHLDB1,* and *TCHHL1* were analyzed in Bangladeshi patients [81]. A *CLDN1* polymorphism was positively associated with early onset of AD in Ethiopian patients [35], but no association was found in the Finnish variants [47]. *CARD11*-R30W has been associated with recurrent infections, autoimmunity, and severe atopy [78], and other dominant, negative mutations in *CARD11*, leading to dominantly inherited, severe atopy have been described in 4 unrelated USA families [79]. Within the same protein family, downregulation of *CARD14* was reported to lead to severe AD and reduced skin protection against infection as well as dysregulated cutaneous inflammation pathways [80]. Rs199691576, a polymorphism of *CP27A1*, was also related to AD in Japan [82].

Mutations in barrier and immune-related genes, i.e., *KLK7, SPINK5, DEFB1, KDR, IL5RA, IL9, IL12RB1*, and *IL13,* were found more frequently in Korean AD patients than in healthy controls [64]. *SPINK5* (serine peptidase inhibitor kazal type 5), a protein involved in epidermal cell differentiation, was also associated with AD in Ethiopian [66] and Japanese patients [49].

Other genes involved in immune functions, such as *IL2RA* [13,76], *IL4* and *ADAM33* [84], *TGFB1* [57], and *MIF* promoter [42] have also been significantly associated to increased risk of AD. The relationship of TLRs polymorphisms with AD, i.e., *TLR2* rs55743708(G>A) and *TLR4* rs4986790(A>G) were reported to increase levels of IL-4 and IL-10 in Russian AD patients [61], while other authors found no association of *TLR2* polymorphisms, i.e., rs5743708 and rs4696480, in Turkish children with AD [69]. Also, significant SNPs in *TSLP*, a lymphopoietin, have been reported in Chinese Han [38], Korean [43], and American [62,71] patients. Lymphotoxin α, a protein involved in IL-2 and IL-4 signaling events, more specifically, *LTA* rs2844484, was associated with AD in Greenland patients [67]. In a study performed in Jordan investigating the relationship between *RETN* gene polymorphisms and AD, rs3745367 was found significantly associated with AD in a gender- and age-specific manner [46]. 

Variants in extracellular matrix genes such as *COL5A3* rs2287807 and *MMP9* rs17575 were found as significantly related to AD in a meta-analysis performed in French, Canadian, and UK families [93]. *COL6A6* minor allele (*AA*) in rs16830494 and the rs59021909 (*TT*) allele and the rs200963433 heterozygous (*CT*) showed higher frequency in patients than in controls, although no statistical significance was reached [83]. *TMEM232* rs11357450 had the strongest association with the risk of AD among all the variants analyzed by Wu et al. [74]. Mutations in *SHARPIN*, a protein involved in epidermis development, that were exclusively present in patients, decreased its expression in AD lesions [55]. *GSDMB* rs921650 was characterized as a strong risk factor for eczema [13].

### 3.5. Epigenetic Studies 

Over the period included in this revision, the studies of epigenetic modifications on atopic dermatitis were focused on DNA methylation and microRNAs. These epigenetic mechanisms have been shown to be crucial regulators in different allergic conditions [100,101], although histone modifications have also been studied in the context of allergic diseases and have been shown to play a role in their development [102]. 

With respect to DNA methylation, two articles studied such modification at a whole-genome level in blood samples. In the first one, Ferreira et al. analyzed the association of DNA methylation with different allergic risk factors. In this manner, they detected 36 genes with DNA methylation sites nearby that were associated with differences in gene expression between allergic patients and healthy controls. Additionally, they found an association between smoking and the methylation state of *PITPNM2* [13], potentially involved in neutrophil function [103,104]. The second study found 490 CpGs differentially methylated between AD patients with eczema herpeticum and healthy controls and 6 CpGs differentially methylated when comparing AD patients without eczema herpeticum and healthy controls. Among these sites, they identified CpG methylation sites in IL4 and IL13, which suggested that there was a significant association between these methylations and the phenotype observed in the patients [28]. Another two studies were centered on the methylation state and its effect on gene expression of *NLRP2* [33] and *SIRL-1* [31]. These studies showed the influence of single nucleotide polymorphisms, a correlation of the gene expression level, and the presence or absence of AD condition.

Regarding the miRNA research, the different analyzed studies can be grouped by two main approaches to the function of miRNAs in the development of AD. On the one hand, the studies that assessed for the upregulation or downregulation of miRNAs in lesioned tissue of AD patients. In this way, several differentially expressed miRNAs were described in AD lesions. When comparing the lesional tissue of AD patients with normal skin samples of healthy controls, Yang et al. described miR-124 downregulation in AD lesional tissue [25] and in an in silico interaction analysis of differentially expressed miRNAs, Li et al. [32] postulated that downregulation of hsa-let-7a-5p would potentially upregulate CCR7, a chemokine receptor involved in the activation of T cells [105]. They also found a differential expression of miR-143, whose potential target is DENND1B, which is involved in the proliferation of T-cells [34]. In addition, the authors suggest that the downregulation of miR-26 would regulate hyaluronan synthase 3 (HAS3), which is upregulated in AD skin [32,106]. After comparing lesional skins samples with non-lesional skin samples in AD patients, Ding et al. proposed a regulatory network of differentially expressed genes that included 182 miRNAs, and among them, hsa-miR-148b, hsa-miR-152, and hsa-miR-324 [24]. Finally, using primary adult human keratocytes, several out of the 372 most common miRNAs were dysregulated when exposed to IL-4, which plays a key role in the development of AD [27].

On the other hand, there are some studies that look for miRNAs differentially expressed in sera from AD patients compared with healthy controls. Thus, the levels of miR-144 detected in umbilical cord serum were higher in those Japanese children that would develop AD at one year of age [26], levels of miR-151a and miR-409 were found to be in higher in sera from Chinese AD patients compared with healthy controls [30], and finally, miR-146a showed no difference in serum levels between patients with AD and healthy individuals [29], despite previously demonstrating a role in the regulation of the immune system and inflammatory responses pathways [107,108]. This second method of retrieving candidate miRNAs may serve as a feasible and less invasive way of obtaining new AD biomarkers, whereas the former procedure in lesioned tissue focused more on the mechanistic of action of such RNAs.

## 4. Discussion

Herein, we have systematically reviewed the literature related to genetics and epigenetics of AD published between June 2016 and December 2019. We have found 58 original articles and 6 meta-analyses and also 9 epigenetic studies. A total of 62 genes have been analyzed in the selected publications, 31 of which had not been reported as potentially associated with AD before June 2016.

One remarkable feature about allergic diseases is the diversity in the potential phenotypes sharing the same genotype, indicating that there appear to be additional components that increase the complexity of the regulation and development of such conditions, or at least shape its evolution over time [109,110]. Epigenetic regulation has emerged as a key factor that was missing to completely understand the molecular basis of allergic disease [111].

### 4.1. Filaggrin

Up to the revision date of this review, *FLG* LoF mutations were the most significantly associated genetic variants for AD. Filaggrin is a key protein in the differentiation of the epidermis and the formation of the skin barrier, which is necessary to prevent water loss through the epidermis and to avoid the entry of allergens, toxins, and pathogens [112]. Its precursor profilaggrin is encoded by the *FLG* gene, which is located on chromosome 1q21.3 [113] within a region known as the epidermal differentiation complex (EDC) comprising over 50 genes encoding proteins involved in terminal differentiation and cornification of keratinocytes [114]. LoF mutations in the exon 3 completely hinder FLG protein expression, increasing the risk of AD [113,115,116,117,118,119]. A meta-analysis of 24 studies on *FLG* mutations determined a 3-fold increased risk of AD in those individuals carrying one or more *FLG* LoF, singling out the influence of one gene in such a heterogeneous disease [120]. More than 300 *FLG* LoF variants have been identified in the *gnomAD* browser, an international database of exome and genome sequencing data (https://gnomad.broadinstitute.org), more than 20 of them associated with susceptibility to AD [85].

The results showing that *FLG* null mutations conferred risk for allergic sensitization and susceptibility to ezcema-associated asthma are well aligned with previous studies [121,122,123,124,125,126,127]. All these findings support the idea that *FLG* mutations lead to functional epidermal barrier defects, increasing skin permeability and subsequent allergic sensitization, promoting the Th2 inflammatory response, and eventually leading to asthma [122]. The “outside–inside” theory of AD pathogenesis proposes that epidermal APCs in AD patients are overexposed to danger signals because of their impaired skin barrier, leading to APC maturation and T-cell-mediated inflammatory skin disease [128]. 

AD has been divided into early-onset and late-onset forms. Early-onset AD would be especially driven by genetic factors, whereas late-onset AD might depend on environmental exposures [129]. Common *FLG* null mutations associated with early-onset AD are described in different populations [126,130,131,132,133]. *FLG* LoF mutations have also associated with moderate-or-severe AD cases [117,122,134,135,136,137,138]. 

The most frequent *FLG* LoF mutations (R501X, 2282del4, S3247X, and R2447X) are present in 7%–10% of Europeans [115,120] while these mutations are rare in Asian patients, who carry specific mutations [45,50,139,140]. Thus, K4022X has been reported as the most prevalent variant in Korean AD patients [50,51,87]. Interestingly, *FLG* mutations in Korean AD patients seem to be less frequent than in other East Asian countries, most likely due to genetic and environmental factors or mutations in other barrier genes [50,87]. Also, the analysis of *FLG* mutations in East Asia showed a geographic distribution in agreement with the history of human migrations [45]. 

On the other hand, *FLG* LoF mutations could be less common in patients with African descent than in those with European or Asian descent [139,140], although other studies have shown that AA children had an increased risk of AD compared with European children [140]. The prevalence of AD in the US was reported as the highest among AA patients, but this population remains largely understudied [141,142]. Recently, two studies using current sequencing methods were able to identify rare *FLG* LoF variants in AA children associated with more persistent AD [85,90]. The prevalence of common *FLG* variants in children of African ancestry is less frequent than those of European or Asian ancestry [85]. Moreover, hygiene habits, vitamin D level, sun exposure, microbiota, genetics, and skin barrier characteristics could also influence the association of ethnicity with AD [1,143]. 

The implementation of new technologies like NGS to analyze cohorts of understudied populations, as well as newer bioinformatic tools, will allow the identification of new *FLG* LoF mutations and confirm the variation among the different ethnicities. 

### 4.2. Other Genes

Regarding research contributions in the period of this review, new components of the extracellular matrix have been described to be associated with AD [64,81,83,93,144]. These new associations highlight the importance of such structure in the development of AD and in the integrity of the skin barrier. Thus, COL5A3, COL6A6, and MMP9 are important for the collagen formation [145,146,147]; KDR, one of the two receptors of the VEGF, has been associated with integrin cell surface interactions with extracellular matrix [148]; mutations in STS (steroid sulfatase) have been associated to X-linked ichthyosis [148]; rare variants of MCM10, a key component of the pre-replication complex, have been described for the first time associated to AD [81]. Regarding TGF-β1 [57], besides its roles in other pathways, different works have shown its role in extracellular matrix assembly and disassembly [149,150]. 

Some genes associated to AD have been related to innate immune system pathways, providing solid evidences of the relationship of the innate immune system with the disease and its progression. On its behalf, most of the genes of the review associated to innate immune pathways (*ADAM33, MIF, MMP9, ORM2, RETN,* and *TLR2*) are related to neutrophil degranulation that contributes to the inflammation of the tissue in the AD [151,152]. In addition, a substantial number of publications [61,69,95,153] emphasize the importance of Toll-like receptor cascades on the development of AD and its link with other allergic diseases [154,155]. The *TLR-2* rs4696480 polymorphism has also been associated with AD severity in adult patients in two different populations, and supported with functional studies [156,157]. However, one of the studies included in this review reported a lack of association of this polymorphism in Turkish children with AD [69], which could be due to the fact that differences in genetics and environmental factors appear to be relevant in the development of allergy. New pieces of evidence have been added to the previously reported association of genes such as *ADAM33*, *CARD11,* and *DEFB1* with AD [64,67,78,79,84]. In this line, *ADAM33* has also been associated to other allergic diseases like asthma [158] and allergic rhinitis [159]. *CARD11* is required for B- and T-cell receptor signal transduction and activation of NF-κB transcription factor [160]. *DEFB1* is an antimicrobial peptide implicated in the resistance of epithelial surfaces to microbial colonization. It is a member of the family of defensins, peptides made by neutrophils, and it has been proposed as a link between the innate and the adaptive immune systems [161,162]. 

It is noteworthy that several of the new genes listed in this revision do not fall into any of these functional groups. This may be due to different causes, as because of little knowledge about some genes or because they are not properly curated yet. This might be the cases of *CARD14*, with similar functions to *CARD11* [163]; *VSTM1*, which behaves as a cytokine [164]; *LILRA6*, a member of the leukocyte immunoglobulin-like receptor family [165]; mutations in *DOCK8* are responsible for an immunodeficiency syndrome [166]; *NLRP2* is involved in inflammatory processes [167,168]; *RTEL1*, a helicase involved in telomere maintenance, has also been found associated to severe dyskeratosis congenita [169]; LT-α (also known as TNF-β), which is a cytokine produced by lymphocytes [170]. Additionally, *ADCY10*, *CUX2*, *MAST2*, *MTF1*, *PANX3*, *PHLDB1,* and *SCAND3* have been associated to AD in a single study of exome sequencing [81]. The S100 fused type protein (SFTP) family includes genes which are mainly expressed in stratified epithelia and play a role in epithelial homeostasis [114]. SFTPs contain two calcium-binding domain EF-hand motifs and are associated with cytoplasmic intermediate filaments as well as minor components of the cornified envelope [114]. This family of proteins include 7 members, FLG being its most studied member and certainly showing an association with AD. Besides FLG, only FLG2 and HRNR were previously associated with AD [171,172,173]. Interestingly, over the last 5 years, other 3 members of the family have been associated with AD. The three members now associated with AD, CRNN, RPTN, and TCHHL1 were known to be involved in different epithelial disorders [174,175,176]. Taken together, SFTP family proteins pose as pivotal players in the proper skin cornification and in the development of AD.

### 4.3. AD Epigenetics

In recent years, the search for risk factors that help to understand how allergic diseases develop has become one of the main objectives of the research. The plasticity observed in the different phenotypes associated to an underlying genotype suggests that additional components may provide complexity to the processes that lead to the development of the disease, or, at least, influence its evolution [109,177]. In the last years, the focus has been set on epigenetic modifications, which can lead to the development of allergic diseases. Epigenetic regulation has emerged as a pivotal key in the comprehension of the molecular basis of allergic conditions [111,178]. 

Most of the research on AD epigenetic regulation has focused on the posttranscriptional regulation mediated by miRNAS. miRNAs constitute a class of small non-coding RNAs, with a size ranging from 17 to 25 nucleotides, and a sequence that allows them to bind to specific mRNAs. This key feature permits the posttranscriptional modulation of targeted genes by triggering mRNA degradation and/or inhibition of translation [179]. According to several functional studies, miRNAs are involved in virtually every cellular process [180]. miRNAs have also been related to immune system regulation, miR-21, miR-146a, and mIR-155 being the most extensively studied. A role in the regulation of the immune response and tissue inflammation in allergic diseases has been shown [101]. 

The analysis of lesional tissues has provided new miRNA molecules that could regulate different mechanisms and signalling pathways that are altered in AD lesions. As a general feature, a decrease of miRNAs involved in the regulation of the immune response and an increase of miRNAs involved in epidermis development is observed in these studies. Thus, downregulation of miR-124 in AD lesions has been shown to control NF-κB-dependent inflammatory responses in keratinocytes and chronic skin inflammation in atopic eczema [25]. Bioinformatics analyses from two studies suggest that miRNAs can influence the transcriptional regulation of signalling pathways related to the synthesis of extracellular matrix components, such as arachidonic acid and hyaluronic acid, as well as participate in processes such angiogenesis, lymphangiogenesis and apoptosis, all of which are involved in AD progression [24,27].

In addition, the use of miRNAs as biomarkers in allergic diseases is increasingly described [181,182]. In this sense, miR-151a and hsa-mir-144-3p have been proposed as potential biomarkers in AD, as they have been shown to be differentially expressed in serum and umbilical cord serum, respectively [26,30]. miR-151a would reduce IL12RB2 levels in T-cells, favouring the increase of Th2 cells, which are central in the pathogenesis of AD [30,183]. Also, an increased expression of miR-144 would reduce *ABCA1* mRNA and protein levels and induce a proinflammatory response via NF-κβ [26]. Nevertheless, the differences in the expression of these small non coding RNAs observed in the lesional skin does not necessarily translate to changes in serum levels, which impairs their use as biomarkers. This is the case of miR-146a, which has been shown to be upregulated in AD lesional skin when compared to healthy controls [184] but showed no differences in serum levels [29]. 

Another extensively studied epigenetic mechanisms is DNA cytosine methylation [185]. This modification occurs in CpG dinucleotides which are grouped in the so called CpG islands, frequently located in intergenic regions, as well as in promoter region of genes [186]. CpG islands methylation of gene promoters is related to the repression of the transcription, i.e., CpG islands of genes that are being actively transcribed do not usually present methylation, while non-transcribed genes present CpG islands with high degrees of methylation [187]. Cytosine methylation suppresses gene transcription, as it causes chromatin condensation and prevents transcription factors from binding to their target sequences in promoters [185]. Different EWAS have shown differential methylation patterns associated with some pathologies [188,189] or even with the exposure to different agents [190,191,192]. Two studies showed gene expression modulation due to exposure to tobacco smoke in AD [13,33]. Thürmann et al. showed that *NLRP2* was differentially methylated, due to both the effect of polymorphisms and tobacco smoke [33], and Ferreira et al. found differences in the methylation of *PITPNM2*, which were partly associated with environmental tobacco smoke [13]. Interestingly, both genes are involved in innate immune responses, with *NLRP2* involved in inflammatory processes related to macrophages [193,194] and *PITPNM2* related to neutrophils [103,104]. Other two studies found differences in DNA methylation patterns in AD patients. The first one found differences in methylation of the promotor region of the VSTM1 gene locus [31], which encodes the protein SIRL-1 that has been proposed to inhibit crucial pro-inflammatory functions in human myeloid cells [195,196]. The second one was a genome wide methylation study that found different patterns of methylation in over 490 sites in AD patients with eczema herpeticum, and where Boorgula et al. identified a significant association between *IL4* and *IL13* methylation and the AD phenotype, as well as with serum IgE levels [28]. However, this methylation patterns where shown to be highly influenced by the eosinophilic count [28]. 

## 5. Final Remarks

In the present article, we have evaluated the last 5 years of AD-related literature using systematic review methodology. We have focused on genetics and epigenetics aspects of the disease, monitoring the different polymorphisms and gene variations associated to the onset or severity of AD, comparing studies performed in different locations and including several ethnicities, therefore showing an up-to-date picture of current knowledge. Some of the retrieved articles used state-of-the-art technology when assessing their findings, including genome-wide sequencing of representative samples of patients. An exhaustive analysis of risk of bias and quality of the 64 selected articles have allowed us to ponder the validity of the reported associations. Another strong point is the inclusion of epigenetic studies.

Regarding limitations to the present review, it has to be point out that we have restricted our analysis to those genes included in the articles published in the last 5 years. Although the main genes related to disease onset and development, i.e. filaggrin, have been included, we are aware that other important genes, already reported elsewhere, may be missing here. Since our goal is to update the topic with new results, we highly recommend the interested reader to consult the previous reviews for more information [16,17].

In addition, we should remark that most genes have been described only once and for a limited number of patients. For instance, *DOCK8* has been identified in a single case report. Larger clinical trials would be required to unambiguously link these genes to AD. The universalization of the whole genome techniques will allow the discovery of new mutations or confirm the already known ones in different populations.

New developments in genetics and epigenetics technology offer opportunities to improve the diagnosis of AD patients, ascribing them to specific genetic groups and allowing the tailoring of therapy with the best response to ensure the most convenient patient care.

## Figures and Tables

**Table 1:** Summary of findings from the selected genetic studies.

Reference	Study Type	Population/Country	Objective	Sample Size	Genes	SNP/Mutation	Results/Conclusion
[77]	WES (Whole-exome sequencing)	Iraq	To determine DOCK8 deficiency	1 child	*DOCK8*	c.3332delT,	Mutation present in hyperimmunoglobulin E syndrome (HIES) and non-Hodgkin lymphoma patient.
						Phe1113Leufs *2 (rs140392509)	
[65]	Candidate gene	Denmark	To determine association of atopic dermatitis (AD) with ichthyosis vulgaris (IV) and actinic keratosis (AK)	481 AK patients, 9112 Healthy controls (HC)	*FLG*	1537C>T(R501X) (rs61816761)	*FLG* homozygous loss of function and AK (in 0.8% of AK studied vs. 0.2% in control population)
						2318_2321del (2282del4)	
						7375C>T (R2447X; rs138726443)	
[66]	Candidate gene	Ethiopia	To elucidate SNVs associated with AD	184 patients of AD and 186 HC	*SPINK5*	rs2303063;	Significant association with AD.
						rs2303067	
[35]	Candidate gene, WES	Ethiopia	To elucidate SNVs associated with AD To establish the role of *CLDN1* variants in Ethiopian AD patients	22 patients for WES; 159 AD patients and 192 HC for genotyping	*CLDN1*	rs17501010	rs893051 is associated with development of AD in early life.
						rs9290927	
						rs9290929	
						rs893051	
[46]	Candidate gene	Jordan	To study the association between *resistin* gene polymorphisms and AD	162 AD patients, 161 HC	*RETN*	SNP +299 G>A (rs3745367)	rs3745367 associated with AD in a gender- and age-specific manner (male, less than 10 y)
						SNP +157 C>T (rs3219177)	
[57]	Candidate gene	Iran	To identify association of SNPs in *IL-10* and *TGF-β1* and AD in Iranian patients	89 children with AD, 138 HC	*TGF-β1*	cdn 10	cdn10/C allele, CC genotype associated with AD
						cdn 25	cdn 25/C allele associated with AD
[68]	Candidate gene	Chinese Han	To identify variants in Chinese Han population associated to AD	4619 AD patients and 10789 HC	*CD207/VAX2*	rs112111458 (allele G/A)	Association of rs112111458 and AD
[69]	Candidate gene	Turkish children	To evaluate if some *TLR2* gene polymorphisms are associated with AD	70 children with AD, 69 HC	*TLR2*	rs5743708 (R753Q)	None
						rs4696480 (A-16934T)	
[70]	Candidate gene	Isle of Wight	To study the association of *FLG* loss of function with atopic march	1150 participants of the Isle of Wight birth cohort	*FLG*	R501X (rs61816761)	*FLG* loss of function mutations are associated with early life eczema at age 1, 2 and 4 years and was consistently associated with rhinitis from 4 years onwards
						2282del	
						S3247X (rs150597413)	
[71]	Candidate genes	USA (mixed population of children)	To determine whether variations in *FLG* and *TSLP* genotype corresponded to differences in therapeutic treatment use over time	842 children with AD	*FLG*	R501X (rs61816761)	Variations in *FLG* and *TSLP* genotype were associated with differences in self-reported skin clearance, TCI usage, and steroid usage
						2282del4	
						R2447X (rs138726443)	
						S3247X (rs150597413)	
					*TSLP*	rs1898671	
						2282del4	

[78]	WES	Canada	To identify the genetic aberration in 4 related patients with combined immunodeficiency, early-onset asthma, eczema, and food allergies, as well as autoimmunity	4 related patients	*CARD11*	hg19:chr7:2987341:G>A NM_032415:exon3:c.C88T:p.R30W (rs145474800)	*CARD11*- R30W is associated with recurrent infections, autoimmunity, and severe atopy. The novel R30W mutations described abrogate the NF-κB pathway and lead to decreased IL-2 and IFN-γ secretion and lymphocyte proliferation
[72]	Candidate gene	Poland	To investigate the importance of 4 common *FLG* null mutations in the susceptibility to eczema in Polish children population	50 children with AD, 37 children with non-atopic eczema and 71 HC children	*FLG*	R501X (rs61816761)	*FLG* null mutations and AD are associated but explain only a part of AD cases (13.8%)
						2282del4	
						R2447X (rs138726443)	
						S3247X (rs150597413)	
[73]	Candidate gene	Different ethnic origins (Dutch, Cape Verdean, Dutch Antillean, Moroccan, Surinamese-Creole, Surinamese-Hindustani, Turkish children)	To study the association of known genetic factors and ethnic origin with the development of eczema	3096 children	*FLG*	2282del4	Carrier frequencies of *FLG* mutations in children of non-Dutch origins were low.
						R2447X (rs138726443)	
						R501X (rs61816761)	
						S3247X (rs150597413)	
[91]	Meta-analysis	Canada (Caucasian)	Study the effect of *FLG* mutations on contact dermatitits (CD)	165 patients with CD, 891 HC	*FLG*	2282del4	Association between *FLG* loss of function mutations and contact polysensitivity, especially in R501X polymorphism.
						R2447X (rs138726443)	
						S3247X (rs150597413)	
						R501X (rs61816761)	
[13]	GWAS (Genome-wide association study)	European ancestry	To identify shared risk variants of a broad allergic disease phenotype that considers the presence of asthma, hay fever and eczema	180,129 cases with asthma and/or hay fever and/or eczema, and 180,709 HC	*FLG*	R501X (rs61816761)	This SNP is a stronger risk factor for eczema than for hay fever or asthma.
					*RPTN-[ ]-HRNR*	rs12123821	This SNP is a stronger risk factor for eczema than for hay fever or asthma.
					*IL1R2-[ ]-IL18R1*	rs12470864	This SNP is a stronger risk factor for eczema than for hay fever or asthma.
					*WDR36-[ ]-CAMK4*	rs6594499	This SNP is a stronger risk factor for hay fever than for eczema or asthma.
					*IL2RA*	rs61839660	This SNP is a stronger risk factor for eczema than for hay fever or asthma.
					*GSDMB*	rs921650	This SNP is a stronger risk factor for eczema than for hay fever or asthma.
[36]	Candidate gene	Russia	To explore the frequency *FLG* mutations and CNVs in AD patients and control subjects of Russian and Tatar ethnic origin living in Volga-Ural region of Russia	177 Russian, 126 Tatar AD patients; and 152 Russian, 109 Tatar HC	*FLG*	2282del4 R501X (rs61816761) R2447X (rs138726443)	Significant differences in 2282del4 frequency were found between Tatar AD patients and HC. The allelic frequency of the R501X mutation in AD patients was 0.85% and in HC -0.47%. The allelic frequency of R2447X was 1.75% in patients, and 1.33% in HC.
[37]	Candidate gene	India	To investigate the personal consequences of having atopic dermatitis and/or hand eczema and *FLG* mutations	163 patients and 86 HC	*FLG*	S2889X (rs782477344)	Mutations in S2889X constituted 96.4% of all *FLG* mutations. No carrier of R501X and Q2417X mutations was identified. *FLG* mutations are associated with irritant contact dermatitis with or without atopy, allergic contact dermatitis without atopy, and idiopathic subtypes. *FLG* mutations were associated with more severe hand eczema.
						2282del4	
						R501X (rs61816761)	
						Q2417X (rs528722713)	
						2282del4	
						R2447X (rs138726443)	
[83]	WES	Korea	To identify family-specific candidate genetic variants associated with early-onset AD in Koreans.	3 families (2 affected AD and 2 unaffected individuals) for WES. 112 AD and 61 HC for validation studies.	*COL6A6*	rs16830494	*COL6A6* variants may be risk factors for AD because the minor allele (AA) in rs16830494 and the rs59021909 (TT) allele and the rs200963433 heterozygous (CT) frequency were all higher in AD cases compared to controls, but no significant association was reached.
						rs59021909	
						rs200963433	
[38]	Candidate gene	Chinese Han	To study in the Chinese Ham population the association AD with previously reported SNPs	3013 AD patients, 5483 HC	*TLR1-TLR6*	rs2101521	SNPs rs2158177 and rs1837253 are associated with AD in Chinese Han population
					*WDR36-CAMK4*	rs1438673	
					*PTGER4*	rs7720838	
					*NFATC2*	rs6021270	
					*IL1RL1-IL18R1*	rs3771175	
					*THSD7B*	rs1469621	
					*RAD50/IL13*	rs2158177	
					*TSLP*	rs1837253	
[39]	Candidate gene	Sweden	To explore the longitudinal relation between preschool eczema, *FLG* mutation, or both and IgE sensitization in childhood.	1890 children	*FLG*	2282del4	Preschool eczema is associated with IgE sensitization to both food allergens and aeroallergens up to 16 years of age. *FLG* mutation is associated with IgE sensitization to peanut but not to other allergens. Sensitized children with preceding PSE are more often polysensitized.
						R501X (rs61816761)	
						R2447X (rs138726443)	
[40]	Candidate gene	USA	To elucidate the associations between *KIF3A* SNPs and asthma, eczema, and AR, alone and in combination.	7000 children and 1020 HC. Results were replicated in 762 children with atopy.	*KIF3A*	rs9784600	*KIF3A* is associated with asthma + eczema. The presence of AR comorbidity did not increase the genetic association of *KIF3A* with asthma or even with asthma+ eczema.
						rs9784675	
						rs11740584	
						rs7737031	
						rs17691077	
						rs2299007	
						rs3798130	
						rs12186803	
						rs1468216	
						rs2023822	
						rs2237059	
						rs2023823	
[84]	Phenome-WAS (Phenome-wide association study)	Turkey	To dissect the role of immunogenetics in allergy and asthma.	974 children	*ADAM33*	rs2787094	rs2280090 was associated with reduced MEF240s (i.e., the ratio of mean expiratory flow after 240 s of hypertonic saline inhalation with respect to the age- and ancestry-matched reference value) and with an increased risk of allergic bronchitis; rs3918396 was associated with wheezing and eczema comorbidity.
						rs543749	
						rs2280090	
						rs2280091	
						rs3918396	
						rs6127096	
						rs511898	
						rs2280090	
						rs3918396	
					*IL4*	rs2243250	rs2243250 is associated with increased FEV240 (forced expiratory flow volume after 240 s of hypertonic saline inhalation).
						rs2070874	
					*CD14*	rs2569190	Associated with asthma.
					*ADRB2*	rs1042713	No association
						rs1042714	
					*IL13*	rs1800925	No association
						rs1295686	
						rs20541	
					*IL4R*	rs1805015	No association
						rs1801275	
					*MS4A2*	rs1441586	No association
						rs569108	
					*SERPINE1*	rs1799768	No association
					*TNF*	rs1800629	No association
[42]	Candidate gene	Korean	To study *MIF* promoter polymorphisms and total plasma IgE in AD Korean patients	178 AD patients, 80 HC	*MIF*	rs755622 (−173 G to C)	*MIF* promoter polymorphisms in the −173 C allele and the MIF C/5-CATT and MIF C/7-CATT haplotypes were significantly associated with an increased risk for AD.
[41]	Candidate gene	Korea	To identify *FLG* SNP variations and evaluated its association with clinical phenotypes, including AD and other parameters.	81 patients	*FLG*	rs71626704	rs71626704 and rs76413899 were significantly associated with a history of asthma and cheilitis. rs62623409 and rs71625199 were associated with sensitization to environmental allergens.
						rs76413899	
						rs62623409	
						rs71625199	
[43]	Candidate gene	Korea	To investigate the association between four possible TSLP polymorphisms and atopic disease in a Korean population.	-	*TSLP*	rs2289276	rs3806932, rs2289276, and rs2289278 are associated with susceptibility of AD. rs3806932, rs3806933, and rs2289276 form one linkage disequilibrium block. The GTT haplotype strongly contributes to atopic march.
						rs2289278	
						rs3806932	
						rs3806933	
[44]	Candidate gene	UK		224 patients and 40 HC	*FLG*	2282del4	Subjects with *FLG*-null mutations have more mature Langerhans cells in non-lesional skin irrespective of whether they have AD.
						R501X (rs61816761)	
						S3247X (rs150597413)	
						R2447X (rs138726443)	
[45]	Candidate gene	Chinese Han/ Singapore, Chinese Han/ Shanghai, Chinese Han/Shanxi, Korean, Japanese/ Kyushu and Japanese/ mainland	To assess the significance of *FLG* mutations as clinical biomarkers in East Asian populations.	1384 patients and 1031 HC	*FLG*	3321delA	c.3321delA was found in all populations. Some mutations showed south-to-north (or north-to-south) distribution gradient: p.K4022X, the most prevalent *FLG* mutation in northern China and Korea, declined in frequency moving southward; in contrast, c.6950del8 (e.g., p.Q2417X, p.E2422X) showed the reverse. p.S2554X/p.S2889X/p.S3296X/Q1701X mutations were Japanese-specific.
						K4022X (rs146466242)	
						6950del8	
						Q2417X (rs528722713)	
						E2422X (rs374588791)	
						S2554X (rs121909626)	
						S2889X (rs782477344)	
						S3296 (rs760426769)	
						Q1701X (rs4547271)	
[92]	Meta-analysis	China, Taiwan, Japan and Saudi Arabia (Asian population) and Poland, Czech R., Macedonia, Egypt (Caucasian)	To study the association between *IL-4*-590C/T polymorphism and AD susceptibility.	923 patients and 1215 HC	*IL-4*	-590C/T	The *IL-4* -590C/T polymorphism may contribute to AD susceptibility in the overall population and children, especially for Asian children.
[89]	CNV analysis	UK	To assess the contribution of *LILR* and *LILRA3* genes CNV to AD	1482 patients from 378 families	*LILR, LILRA3*		The transmission of one copy of *LILRA6* within families was potentially related to the development of AD.
[47]	Candidate gene	Finland	To test the association of the 4 most prevalent European *FLG* null mutations, the 2 Finnish enriched *FLG* null mutations, the *FLG* 12-repeat allele, and 50 additional epidermal barrier gene variants, with risk of AD, disease severity, clinical features, risk of other atopic diseases, age of onset, and treatment response.	501 patients with AD and 1710 HC	*FLG*	R501X (rs61816761)	2822del4 was significantly associated with early-onset AD and asthma. R501X was associated with early-onset and suggestively with keratosis pilaris. R2447X showed suggestive association with early-onset AD. Baseline IgE values were higher in patients with *FLG* null mutations, but the association was not significant; *FLG* null mutations were not associated with atopic hand eczema, dermographism, or HSV infections.
						R2447X (rs138726443)	
						S3247X (rs150597413)	
						S1020X (rs200360684)	
						V603M (rs2306942)	
						rs12730241	
					*CLDN1*		No significant association with AD
					*CLDN4*		
					*CLDN20*		
					*CLDN23*		
					*OCLN*		
					*IVL*		
					*FLG2*		
					*LOR*		
					*JAM-1*		
					*TJP1*		
[79]	WES	USA	To study *CARD11* mutations in four families with recalcitrant, severe atopic disease.	8 patients	*CARD11*	L194P	The study describes rare hypomorphic dominant negative mutations in *CARD11* in 4 unrelated families, which lead to dominantly inherited, severe atopy, with variable infection beyond the skin.
						R975W (rs1064795307)	
						E57D	
						dup183_196	
[5]	GWAS	UK	To test whether genetically lowered vitamin D levels were associated with risk of asthma, atopic dermatitis, or elevated serum IgE levels.	33996 children	*DHCR7*	rs12785878	No association
					*CYP2R1*	rs10741657	
					*CYP24A1*	rs6013897	
[48]	Candidate gene	Italy	To evaluate the role of *FLG* polymorphisms expression and risk of developing a concomitant *Molluscum contagiosum* sustained skin infection in the pediatric population with AD.	100 children with AD and 97 healthy children	*FLG*	rs79808464	*FLG* mutations are associated with early onset of AD, more severe clinical course of disease, and a significantly increased risk of *M. contagiosum* sustained skin infection
						rs116222149	
						rs11584340	
						rs113136594	
						rs145828067	
						rs374910442	
						rs747005144	
						rs145627745	
						rs144209313	
						rs74129443	
						rs192455877	
						rs150957860	
						rs138055273	
						rs147472105	
						rs183942200	
						rs558269137	
[86]	NGS (Next-generation sequencing)	German	To identify disease association in the locus 11q13.5 using combination of sequencing and functional annotation.	31 AD patients	*LRRC32*	A407T	Association of low-frequency and rare missense mutations within the *LRRC32* gene with AD.
						R518W (rs142940671)	
						R312	
						S411R (rs201424816)	
						R414W (rs201431152)	
						R652C	
[93]	Meta-analysis	French, French- Canadian and UK	To detect new interacting genes involved in eczema	388 French families. Replication in 253 French-Canadian and 207 UK family datasets.	*COL5A3*	rs2287807	Identified significant interaction between two new genes, *COL5A3* and *MMP9*, which may be accounted for by a degradation of *COL5A3* by *MMP9* influencing eczema susceptibility.
					*MMP9*	rs17576	
[85]	NGS	USA	To evaluate *FLG* LoF variation in children of African ancestry and the association with AD and AD persistence.	262 African American children and 133 Caucasians	*FLG*	R501X (rs61816761)	Rare *FLG* LoF variants in African American children are associated with AD and more persistent AD. In contrast to Europeans, no *FLG* LoF variants predominate in African American children. The most common variants were R501X, S3316X, and R826X.
						S3316X (rs149484917)	
						R826X (rs115746363)	
						R2447X (rs138726443)	
						Q570X (rs192402912)	
						R3409X (rs201356558)	
						S3247X (rs150597413)	
						Q3818X (rs148606936)	
						H440fs	
[90]	CNV (Copy number variations) analysis	African American (USA)	To study *FLG* LoF and CNV in African American population	39 children with AD	*FLG*	R501X (rs61816761)	rs149484917 is a population-specific *FLG* LoF unique to several populations of African Ancestry. Two new *FLG* LoF were identified (488delG and S3101X)
						R826X (rs115746363)	
						S3316X (rs149484917)	
						488delG	
						S3101X	
[49]	Candidate gene	Japan	To study polymorphisms of SPINK5 gene in Japanese AD patients	57 patients, 50 HC	*SPINK5*	Q267R (rs6892205)	Only S368N frequency differed between Japanese patients and data from Human Genetic Variation Database. Algorithms predicting functional effects of amino acids substitution showed significant scores for R654H
						A335V (rs34482796)	
						S368N (rs230306)	
						D386N (rs2303064)	
						R711Q (rs3777134)	
						E825D (rs2303070)	
[50]	Candidate gene	Korea	To examine the spectrum of null-mutations and compare with other Asian countries	70 patients	*FLG*	R501X (rs61816761)	Only 11 AD patients had *FLG* mutations. This frequency was lower than that described for other Asian populations (Chinese, Japanese, Singaporean)
						3321delA	
						Y1767X (rs1222103354)	
						S1695X (rs772851618)	
						Q1701X (rs145738429)	
						Q1745X (rs1209640261)	
						Q1790X (rs200622741)	
						S2554X (rs121909626)	
						S2889X	
						S3296X (rs761212672)	
						K4022X (rs146466242)	
						3222del4	
						S1515X (rs180768115)	
						Q2417X (rs528722713)	
[51]	Candidate gene	Korea	To investigate the genetic polymorphisms of *FLG* in Korean AD patients	9 ichtyosis vulgaris patients 50 AD patients 55 HC	*FLG*	K4022X (rs146466242)	This loss-of-function mutation was only found in AD patients. 62 new SNPs were identified
[87]	NGS	Korea	To investigate clinical characteristics of AD patients with *FLG* mutations. To determine differences between patients with and without *FLG* mutations	1110 patients, 68 with mutations in *FLG* gene	*FLG*	K4022X (rs146466242)	Null alleles were associated with early onset of AD and higher risk of developing the disease by age 2 years.
						3321delA	EASI score was also higher in these patients
[76]	GWAS	UK, Netherlands	To investigate longitudinal phenotypes of AD in two independent cohorts	UK: 9894 individuals (ALSPAC) NL: 3652 individuals (PIAMA)	*INPP5D*	rs1057258-c	Six classes based on temporal trajectories of rash were identified: persistent, early-onset/late resolving, early-onset/early-resolving, medium-onset/resolved, late-onset/resolved. *FLG* null mutations were strongly associated with early-onset and late-onset of AD (*p* < 0.00001: ALSPAC) and early-onset/late-resolving (*p* = 0.0006: PIAMA)
					*PRR5L*	rs12295535-t	
					*STAT3*	rs17881320-t	
					*PPP2R3D*	rs2143950-t	
					*ACTL9*	rs2164983-a	
					*IL6R*	rs2228145-c	
					*KIF3A*	rs2897442-c	
					*OVOL1*	rs479844-g	
					*C11orf30*	rs7927894-t	
					*IL22*	rs2227482-t	
					*IL21*	rs17389644-a	
					*IL2RA*	rs6602364-g	
[80]	WES	USA (Hispanic, Caucasian, African-American)	To identify rare DNA variants conferring significant risk for AD	3 patients	*CARD14*	c.1778T>C, I593T	Downregulation of *CARD14* led to severe AD and reduced skin protection against infection and dysregulated cutaneous inflammation pathways
						c.2206A>C,N737H (rs535171797)	
[81]	WES, rare enrichment analysis	Bangladeshi	To analyze the genetic architecture of patients with AD from a Bangladeshi community in London, UK	53 cases and 42 HC from 70 families	*SCAND3*		Some rare sequence variations of candidate genes have been identified. *FLG* loss-of-function variations were carried by almost 50% of AD-affected individuals.
					*TCHHL1*		
					*ADCY10*		
					*MTF1*		
					*MCM10*		
					*ORM2*		
					*CUX2*		
					*MAST2*		
					*PHLDB1*		
					*FLG*		
[52]	Candidate gene	Poland	To explore the role of different SNPs at 11q13.5 in predisposing to allergic phenotypes	270 AD patients, 540 HC		rs7927894	The haplotype TATG in these SNPs fully explained the association with AD (*p* = 0.00021)The TG haplotype in the last two SNPs was also related to allergic rhinitis
						rs2513517	
						rs7930794	
						rs7125552	
[94]	Meta-analysis	Asian, Caucasian	To assess the genetic relationship between Il-10 polymorphisms and susceptibility to AD.	16 case-control studies	*IL-10*	*IL-10* -1082a/G	These polymorphisms showed a weak association with AD susceptibility
						*IL-10* -819T/C	
						*IL-10* -592a/C	
[53]	Candidate gene	Netherlands (Caucasians, Asians, Afro- Caribbean)	To investigate whether *FLG* mutations influence the outcome of immunosuppressive therapy	42 patients with severe AD: 3 Asians, 1 Afro-Caribbean, 38 Caucasians	*FLG*	R501X (rs61816761)	*FLG* mutation group showed a trend towards less improvement in the course of 24 weeks of treatment with methotrexate and azothiopine
						2282del4	
						R2447X (rs138726443)	
						S3247X (rs150597413)	
						5321delA	
[54]	Candidate gene	Japan	To elucidate the effect of bi-allelic *FLG* mutation on AD incidence and severity	6 individuals from 3 families	*FLG*	Q1790X+S3296X	The most severe AD was associated with c.3321delA+S2889X bi-allelic combination. By contrast, individuals with S2889X+S3296X did not develop AD
						Q1790X+S2889X	
						S2889X+S3296X	
						Q1701X+S2889X	
						3321delA+S2889X	
[67]	Candidate gene	Inuit	To study the effect of environment on genotype-phenotype association in a genetically homogeneous population, living in two separate areas	615 Greenlandic Inuit individuals 643 Danish Inuit individuals	*ADAM*	rs612709	*LT*α rs2844484 was associated with AD in Greenland patients (*p* = 0.035) The risk of AD was related to the genotype distribution of this SNP, with a significant interaction with the place of residence
						rs528557	
						rs44707	
						rs2787094	
					*ALOX5*	rs4986832	
						rs892690	
						rs2115819	
					*LT-α*	rs2844484	
						rs909253	
						rs1041981	
					*LTC4S*	rs730012	
					*NOS I*	rs7977109	
					*ORMDL3*	rs4065275	
						rs12603332	
					*TBXA2R*	rs4523	
					*TNF-α*	rs1799964	
						rs1800630	
						rs1800629	
[82]	WES	Japan	To investigate rare genetic variants associated with AD	469 AD patients, 935 HC	*APOB*	rs145862664	Gene polymorphism in *CYP27A1*, a gene involved in vitamin D3 metabolism, was related to AD
					*CYP27A1*	rs199691576	
					*C3orf15*	rs193146105	
					*GAK*	rs142107211	
					*VNN2*	rs200230703	
					*USP35*	rs200193128	
					*ZNF749*	rs76428401	
[55]	Candidate gene	China	To investigate the potential role of *SHARPIN* in the pathogenesis of AD	65 AD patients, 100 HC	*SHARPIN*	g.480G>A	SNPs g.4320, g.4334, g.4343, g.4344, g.5363 were present both in patients and controls. The mutations in *SHARPIN* were only present in AD patients, decreasing the expression in AD lesions.
						g.4576A>T	
						g.5070C>T	
						rs200698932	
						rs769935596	
						rs760937092	
						rs1280524235	
						g.4320	
						g.4334	
						g.4343	
						g.4344	
						g.5363	
[56]	Candidate gene	Japanese, Korean	To determine prevalence of *FLG* mutation in AD and IV patients in Japan and South Korea	Japan: 26 IV patients, 91 AD patients South Korea: 76 AD patients	*FLG*	R501X (rs61816761)	Mutation S3296X only appeared in Japanese AD patients. R501X and R826X only appeared in IV patientsThe rest of the mutations were found in both Korean and Japanese patients.
						S2554X (rs121909626)	
						S2889X	
						G1109EfsX (rs133912394)	
						K4022X (rs146466242)	
						S1695X (rs772851618)	
						Q1701X (rs145738429)	
						R826X (rs115746363)	
[58]	Candidate gene	Denmark	To examine the association between loss-of-function mutations in *FLG* and AD and asthma in adult twins	575 adults twins with asthma	*FLG*	R501X (rs61816761)	Within the dizygotic twin population, 11 pairs were discordant for *FLG* mutation. The risk of AD increased in the twin with *FLG* mutation. No significant association was found with *FLG* mutations and asthma
						2282del4	
						R2447X (rs138726443)	
[59]	Candidate gene	Denmark	To explore heritable, environmental, and clinical factors related to persistent AD	417 children: 186 patients (40 with persistent AD), 231 HC. Follow-up study from birth to age 13 y	*FLG*	R501X (rs61816761)	29% of patients with persistent AD had *FLG* mutations. The higher AD genetic risk score, the higher risk of persistent AD.
						2282del4	
						R2447X (rs138726443)	
						S3247X (rs150597413)	
					*FLG2*		
					*SPRR3*		
[60]	Candidate gene	Poland	To identify new potential markers of AD	159 AD patients, 108 HC	*RPTN*	rs284544	In *FLG* WT patients, *RPTN* rs3001978CC was significantly associated with AD early age onset (*p* = 0.033), pruritus (*p* = 0.021), severity of AD (*p* = 0.045) and concomitant asthma (*p* = 0.041) rs 941934 allele *A* was more frequent in AD patients (*p* = 0.007), the homozygous *AA* only appeared in AD patients (*p* = 0.019). The association depended on *FLG* mutations.
						rs28441202	
						rs3001978	
						rs12117644	
					*CRNN*	rs941934	
					*FLG*	R2447X (rs138726443)	
						S3247X (rs150597413)	
[61]	Candidate gene	Russia	To determine the relationship of *TLR*s polymorphisms with AD	25 AD patients, 25 AD and rhinitis/asthma patients, 100 HC	*TLR2*	rs55743708 (G>A)	Increased levels of IL-4 and IL-10. Dysfunction of cell activation.
					*TLR4*	rs4986790(A>G)	Increased levels of IL-4 and IL-1. Weaker cell response to microbial antigens.
[62]	Candidate gene	USA	To examine the effect of *FLG* mutations and *TSLP* (thymic stromal lymphopoietin) polymorphisms on the age of AD onset	822 children (age 2–17 y)	*FLG*	R501X (rs61816761)	*FLG* null mutations were associated with early onset of AD. Number of mutations was associated with timing of onset. No association was found between TSLP polymorphism and timing of onset.
						2282del4	
						R2447X (rs138726443)	
						S3247X (rs150597413)	
					*TSLP*	rs1898671	
[63]	Candidate gene	Taiwan	To investigate the association between gene–environmental interaction and childhood AD	839 mother–child pairs	*GST*	*GST*-T1/M1 mutants	*GST* null genotypes in association with high levels of perfluoroalkyl substances in blood increased the risk of developing AD
[88]	NGS	Singapore population: Chinese, Indian, Malay	To sequence the entire *FLG* coding region in Singaporeans from different ethnicities	334 patients with AD and/or IV	*FLG*	S1515X (rs180768115)	A new technology that improved accuracy and cost-effectiveness is described. New mutations have been identified
						E2422X (rs374588791)	
						S406X (rs189114758)	
						c.6950-6957del8	
						c.1640delG	
						Q368X (rs746899204)	
						3321delA	
						7945delA	
						Q2417X (rs528722713)	
						2952delC	
						9040-9058dup19	
						Q1790X (rs200622741)	
						S1302X (rs754812742)	
						S1515X (rs180768115)	
						4004del2	
						2282del4	
						R2447X (rs138726443)	
						477insA	
						678delA	
						S378X (rs755134998)	
						3036delT	
						10866delA	
						rs10067777	
[74]	GWAS	China	To identify AD susceptibility genes in 5q22.1 and observe expression in AD tissues	3031 cases, 5075 HC	*TMEM232*	rs7701890	rs11357450 had the strongest association with risk of AD (OR = 1.20; *p* = 0.04)
						rs13360927	
						rs13361382	
						rs5870408	
						rs1400764268	
						rs35639206	
						rs137936676	
						rs10617471	
						rs11357450	
[64]	Candidate gene	Korea	To identify mutations and SNPs in barrier- or immune-related genes	279 AD patients, 224 HC	*KLK7*	*KLK7 MT*	More frequent in AD patients (*p* = 0.04). No differences between AD groups
					*FLG*	3321delA	More frequent in moderate/sever AD patients (*p* = 0.038)
						K4022X (rs14646429)	More frequent in patients but not statistically different
					*SPINK5*	1156	More frequent in AD vs HC (*p* < 0.001). No differences between AD groups
						1188	
						2475	
					*DEFB1*	rs5743399	More frequent in AD vs HC (*p* < 0.002). No differences between AD groups
					*KDR*	rs2305948	More frequent in AD vs HC (*p* = 0.03). No differences between AD groups
					*IL5RA*	rs334809	More frequent in AD vs HC (*p* < 0.001). More frequent in mild AD vs moderate/severe
					*IL9*	rs31563	More frequent in AD vs HC (*p* < 0.001). No differences between mild AD and HC; and between moderate AD vs. HC
					*IL12RB1*	rs393548	More frequent in AD vs HC (*p* = 0.02).
						rs436857	More frequent in AD vs HC (*p* = 0.01). More frequent in mild AD vs. HC. No differences between moderate AD and HC
					*IL13*	rs20541	Heterozygous less frequent in patients vs. HC
[95]	Meta-analysis	Germany, Turkey, Italy, Finland, Ukraine, Russia	To assess whether *TLR*s polymorphisms are associate with risk of AD	*TLR2*: 9 studies; 733 cases, 807 HC	*TLR2*	rs5743708	Increased risk of AD for GA heterozygous
				*TLR4*: 6 studies; 646 cases, 601 HC	*TLR4*	rs4986790	AG showed some correlation with risk of AD, but was non-conclusive.
[96]	Meta-analysis	Saudi Arabia, Iran, India, Netherlands, Italy, Poland, Macedonia, Czech Republic, China, Korea, Taiwan, Germany, UK	To summarize the current evidence on association between *IL-10* polymorphisms and susceptibility to AD	15 case-control studies, 1647 patients, 2031 HC	*IL-10*	*IL-10*-1092 G/A	Increased risk of AD associated with IL-10-819 G/A mutation in Caucasian subjects and with *IL-10*-1092 G/A in Asian patients
						*IL-10*-592 A/C	
						*IL-10*-819 G/A	
[75]	GWAS	UK	To assess whether *FLG* expression in umbilical cord blood associates with and predicts AD	94 infants	*FLG*	S3247X (rs150597413)	*FLG* expression in cord blood correlated with AD risk. 2.94-increased risk for mutated *FLG* variants
						2282del4	

**Table 2:** Summary of findings from the selected epigenetic studies.

Cell/Ttissue Types	Epigenetic Assay	Significant Findings	Reference
Primary adult human keratocytes	miFinder miRNA PCR Array	Broad dysregulation of miRNAs upon IL-4 treatment	[27]
Whole blood samples	DNA methylation profiling	Identifying CpG methylation sites in IL4 and IL13 associated with AD phenotype	[28]
Serum	Real-time PCR for miR-146a	miR-146a levels are unaltered in AD patients	[29]
Serum	RNA sequencing	miR-151a and miR-409 overexpressed in Chinese AD patients	[30]
AD lesioned skin	Microarray expression data from GSE32924	Regulatory network comprising 182 miRNAS	[24]
Umbilical cord serum	Exiqon Serum/Plasma Focus micro- RNA PCR Panel (179 miRNAs)	miR-144 levels are higher in the umbilical cord of AD children	[26]
Whole blood samples	DNA methylation profiling	Association between smoking and the methylation state of *PITPNM2*	[13]
Monocytes and neutrophils	*VSTM1* methylation	Polymorphism dependent *VSTM1* methylation	[31]
AD lesioned skin	Microarray miRNA expression (GSE31408)	Hsa-let-7a-5p, has-miR-26a-5p and has-miR-143-3p differentially expressed in lesioned tissues	[32]
Whole blood samples	*NLRP2* methylation	*NLRP2* methylation is associated both to the environment and SNP	[33]
AD lesioned skin	Real-time PCR assay	miR-124 downregulated in AD lesional tissue	[25]

## Supplementary Materials

The following are available online at https://www.mdpi.com/2073-4425/11/4/442/s1, Figure S1: Reactfoam of pathways overview of the genes reported in the selected studies; Table S1: Analysis of the risk of bias for the selected genetic studies; Table S2: Quality assessment of the selected genetic studies.
