# Supplementary material for: Genetics and Epigenetics of Atopic Dermatitis: An Updated Systematic Review
Source: Genes (Basel). 2020 Apr 18;11(4):442. doi: 10.3390/genes11040442 (PMC7231115; doi:10.3390/genes11040442)
Supplement: Supplementary file 1 [file genes-11-00442-s001.zip › Table S1.docx]

Table S1: Analysis of the risk of bias for the selected genetic studies

| **Reference** | **Randomization process** | **Deviations from intended interventions** | **Missing outcome data** | **Measurement of the outcome** | **Selection of the reported result** | **Overall Bias** |
| --- | --- | --- | --- | --- | --- | --- |
| Asad et al., 2016 [35] | High | Low | Low | Low | Low | Some concerns |
| Al-Kzayer et al., 2019 [77] | High | High | High | High | Some concerns | High |
| Andersen et al., 2017 [65] | Some concerns | Low | Low | Low | Low | Some concerns |
| Asad et al., 2019 [66] | Low | Low | Low | Low | Low | Low |
| Banihani et al., 2018 [46] | Low | Low | Low | Low | Low | Low |
| Behniafard et al., 2018 [57] | Low | Low | Low | Low | Low | Low |
| Cai et al., 2017 [68] | Low | Low | Low | Low | Low | Low |
| Can et al., 2017 [69] | Low | Low | Low | Low | Low | Low |
| Chan et al.,2018 [70] | Low | Low | Low | Low | Low | Low |
| Chang et al., 2017 [71] | High | Some concerns | Low | Low | Low | High |
| Dadi et al., 2018 [78] | High | Low | Low | Low | Low | High |
| Debinska et al., 2017 [72] | Low | Low | Low | Low | Low | Low |
| Elbert et al., 2016 [73] | Low | Low | Low | Low | Low | Low |
| Elhaji et al., 2019 [91] | Low | Low | Low | Low | Low | Low |
| Ferreira et al., 2017 [13] | Low | Low | Low | Some concerns | Low | Some concerns |
| Gimalova et al., 2017 [36] | Low | Low | Low | Low | Low | Low |
| Handa et al., 2019 [37] | Low | Low | Low | Low | Low | Low |
| Heo et al., 2017 [83] | High | Low | Low | Low | Low | High |
| Jiang et al., 2018 [38] | Low | Low | Low | Low | Low | Low |
| Johansson et al., 2017 [39] | Low | Low | Low | Low | Low | Low |
| Johansson et al., 2017 [40] | Low | Low | Low | Low | Low | Low |
| Karaca et al., 2016 [84] | Low | Low | Low | Low | Low | Low |
| Kim et al., 2016 [42] | Low | Low | Low | Low | Low | Low |
| Kim et al., 2017 [41] | Low | Low | Low | Low | Low | Low |
| Ko et al., 2018 [43] | Some concerns | High | Low | Low | Low | High |
| Leitch et al., 2016 [44] | Low | Low | Low | Low | Low | Low |
| Li et al., 2016 [45] | Low | Low | Low | Low | Low | Low |
| Liang et al., 2017 [92] | Low | Low | Low | Low | Low | Low |
| Lopez-Alvarez et al., 2016 [89] | Low | Low | Low | Low | Low | Low |
| Luukkonen et al., 2017 [47] | Low | Low | Low | Low | Low | Low |
| Ma et al., 2017 [79] | High | High | Low | Low | Low | High |
| Manousaki et al., 2017 [5] | Some concerns | Low | Low | Low | Low | Low |
| Manti et al., 2017 [48] | Low | Low | Low | Low | Low | Low |
| Manz et al., 2016 [86] | Low | Low | Low | Low | Low | Low |
| Margaritte-Jeannin et al., 2018 [93] | Low | Low | Low | Low | Low | Low |
| Margolis et al., 2018 [85] | Some concerns | Low | Low | Low | Low | Some concerns |
| Mathyer et al., 2018 [90] | High | Low | Low | Low | Low | High |
| Morizane et al., 2018 [48] | Low | Low | Low | Low | Low | Low |
| On et al., 2017 [50] | High | High | Low | Low | Low | High |
| Park et al., 2016 [51] | Low | Low | Low | Low | Low | Low |
| Park et al., 2016 [87] | High | High | Low | Low | Low | High |
| Paternoster et al., 2018 [76] | Low | Low | Low | Low | Low | Low |
| Peled et a., 2018 [80] | High | High | Low | Low | Low | High |
| Pigors et al., 2018 [81] | Low | Low | Low | Low | Low | Some concerns |
| Poninska et al., 2017 [52] | Low | Low | Low | Low | Low | Low |
| Qi et al, 2019 [94] | Low | Low | Low | Low | Low | Low |
| Roekevisch et al., 2017 [53] | Some concerns | High | Low | Low | Low | High |
| Sekiya et al., 2017 [54] | High | High | Low | Some concerns | Low | High |
| Song et al., 2017 [67] | Low | Low | Low | Low | Low | Low |
| Suzuki et al., 2016 [82] | High | Low | Low | Low | Low | High |
| Tang et al., 2018 [55] | Low | Low | Low | Low | Low | Low |
| Teye et al., 2017 [56] | High | High | Low | Low | Low | High |
| Thomsen et al., 2016 [58] | High | High | Low | Some concerns | Some concerns | High |
| Thorsteinsdottir et al., 2019 [59] | Low | Low | Low | Low | Low | Low |
| Trzeciak et al., 2017 [60] | Low | Low | Low | Low | Low | Low |
| Tyurin et al., 2017 [61] | Low | Low | Low | Low | Low | Low |
| Wan et al., 2017 [62] | Some concerns | High | Low | Low | Low | High |
| Wen et al., 2019 [63] | Low | Low | Low | Low | Low | Low |
| Wong et al., 2018 [88] | Some concerns | Low | Low | Low | Low | Some concerns |
| Wu et al., 2017 [74] | Low | Low | Low | Low | Low | Low |
| Yoon et al., 2018 [64] | Low | Low | Low | Low | Low | Low |
| Zhang et al., 2019 [95] | Low | Low | Low | Low | Low | Low |
| Zhao et al., 2019 [96] | Low | Low | Low | Low | Low | Low |
| Ziyab et al., 2017 [75] | Some concerns | Low | Low | Low | Low | Some concerns |
