# Supplementary material for: Genetics and Epigenetics of Atopic Dermatitis: An Updated Systematic Review
Source: Genes (Basel). 2020 Apr 18;11(4):442. doi: 10.3390/genes11040442 (PMC7231115; doi:10.3390/genes11040442)
Supplement: Supplementary file 1 [file genes-11-00442-s001.zip › Table S2.docx]

Table S2: Quality assessment of the selected genetic studies

| **Reference** | **Adequate case definition** | **Representativeness** | **Control selection** | **Control definition** | **Comparability** | **Exposure** | **Method of ascertainment** | **Non-response rate** | **Total score** | **Quality** |
| --- | --- | --- | --- | --- | --- | --- | --- | --- | --- | --- |
| Asad et al., 2016 [35] | 1 | 1 | 2 | 1 | 1 | 1 | 1 | 1 | 9 | High |
| Al-Kzayer et al., 2019 [77] | 0 | 0 | 0 | 0 | 0 | 1 | 0 | 0 | 1 | Low |
| Andersen et al., 2017 [65] | 1 | 1 | 1 | 1 | 1 | 1 | 1 | 1 | 8 | High |
| Asad et al., 2019 [66] | 1 | 1 | 1 | 1 | 1 | 1 | 1 | 1 | 8 | High |
| Banihani et al., 2018 [46] | 1 | 1 | 1 | 1 | 1 | 1 | 1 | 1 | 8 | High |
| Behniafard et al., 2018 [57] | 1 | 1 | 1 | 1 | 1 | 1 | 1 | 1 | 8 | High |
| Cai et al., 2017 [68] | 1 | 1 | 1 | 1 | 1 | 1 | 1 | 1 | 8 | High |
| Can et al., 2017 [69] | 1 | 1 | 1 | 1 | 1 | 1 | 1 | 1 | 8 | High |
| Chan et al.,2018 [70] | 1 | 1 | 1 | 1 | 1 | 1 | 1 | 1 | 8 | High |
| Chang et al., 2017 [71] | 1 | 1 | 0 | 0 | 0 | 1 | 0 | 1 | 4 | Medium |
| Dadi et al., 2018 [78] | 0 | 0 | 0 | 0 | 0 | 0 | 0 | 0 | 0 | Low |
| Debinska et al., 2017 [72] | 1 | 1 | 1 | 1 | 1 | 1 | 1 | 1 | 8 | High |
| Elbert et al., 2016 [73] | 1 | 1 | 1 | 0 | 1 | 1 | 1 | 1 | 7 | High |
| Elhaji et al., 2019 [91] | 1 | 1 | 1 | 1 | 1 | 1 | 1 | 1 | 8 | High |
| Ferreira et al., 2017 [13] | 1 | 1 | 1 | 1 | 1 | 1 | 1 | 1 | 8 | High |
| Gimalova et al., 2017 [36] | 1 | 1 | 1 | 1 | 1 | 1 | 1 | 1 | 8 | High |
| Handa et al., 2019 [37] | 1 | 1 | 1 | 1 | 1 | 1 | 1 | 1 | 8 | High |
| Heo et al., 2017 [83] | 1 | 1 | 1 | 1 | 1 | 1 | 1 | 1 | 8 | High |
| Jiang et al., 2018 [38] | 1 | 1 | 1 | 1 | 1 | 1 | 1 | 1 | 8 | High |
| Johansson et al., 2017 [39] | 1 | 1 | 1 | 1 | 1 | 1 | 1 | 1 | 8 | High |
| Johansson et al., 2017 [40] | 1 | 1 | 1 | 1 | 1 | 1 | 1 | 1 | 8 | High |
| Karaca et al., 2016 [84] | 1 | 1 | 1 | 1 | 1 | 1 | 1 | 1 | 8 | High |
| Kim et al., 2016 [42] | 1 | 1 | 1 | 1 | 1 | 1 | 1 | 1 | 8 | High |
| Kim et al., 2017 [41] | 1 | 1 | 0 | 0 | 0 | 0 | 1 | 1 | 4 | Medium |
| Ko et al., 2018 [43] | 1 | 1 | 1 | 1 | 1 | 1 | 1 | 1 | 8 | High |
| Leitch et al., 2016 [44] | 1 | 1 | 1 | 1 | 1 | 1 | 1 | 1 | 8 | High |
| Li et al., 2016 [45] | 1 | 1 | 1 | 1 | 1 | 1 | 1 | 1 | 8 | High |
| Liang et al., 2017 [92] | 1 | 1 | 1 | 1 | 1 | 1 | 1 | 1 | 8 | High |
| Lopez-Alvarez et al., 2016 [89] | 0 | 0 | 0 | 0 | 0 | 0 | 1 | 0 | 1 | Low |
| Luukkonen et al., 2017 [47] | 1 | 1 | 0 | 0 | 0 | 1 | 0 | 0 | 3 | Low |
| Ma et al., 2017 [79] | 1 | 1 | 1 | 1 | 1 | 1 | 1 | 1 | 8 | High |
| Manousaki et al., 2017 [5] | 1 | 1 | 0 | 0 | 0 | 0 | 0 | 0 | 2 | Low |
| Manti et al., 2017 [48] | 1 | 1 | 1 | 1 | 1 | 1 | 1 | 1 | 8 | High |
| Manz et al., 2016 [86] | 1 | 1 | 1 | 1 | 1 | 1 | 1 | 1 | 8 | High |
| Margaritte-Jeannin et al., 2018 [93] | 1 | 1 | 0 | 0 | 0 | 1 | 0 | 0 | 3 | Low |
| Margolis et al., 2018 [85] | 1 | 1 | 1 | 1 | 1 | 1 | 1 | 1 | 8 | High |
| Mathyer et al., 2018 [90] | 0 | 1 | 0 | 0 | 0 | 1 | 0 | 1 | 3 | Low |
| Morizane et al., 2018 [48] | 1 | 1 | 1 | 1 | 1 | 1 | 1 | 1 | 8 | High |
| On et al., 2017 [50] | 1 | 1 | 1 | 1 | 1 | 1 | 1 | 1 | 8 | High |
| Park et al., 2016 [51] | 1 | 1 | 1 | 1 | 0 | 1 | 1 | 1 | 7 | High |
| Park et al., 2016 [87] | 0 | 0 | 0 | 0 | 0 | 0 | 1 | 0 | 1 | Low |
| Paternoster et al., 2018 [76] | 1 | 1 | 1 | 1 | 1 | 1 | 1 | 1 | 8 | High |
| Peled et a., 2018 [80] | 1 | 1 | 1 | 1 | 1 | 1 | 1 | 1 | 8 | High |
| Pigors et al., 2018 [81] | 0 | 0 | 0 | 0 | 1 | 1 | 0 | 1 | 3 | Low |
| Poninska et al., 2017 [52] | 0 | 0 | 0 | 0 | 1 | 1 | 0 | 1 | 3 | Low |
| Qi et al, 2019 [94] | 1 | 1 | 1 | 1 | 1 | 1 | 1 | 1 | 8 | High |
| Roekevisch et al., 2017 [53] | 1 | 1 | 1 | 1 | 1 | 1 | 1 | 1 | 8 | High |
| Sekiya et al., 2017 [54] | 1 | 1 | 1 | 1 | 1 | 1 | 1 | 1 | 8 | High |
| Song et al., 2017 [67] | 0 | 0 | 0 | 0 | 1 | 0 | 0 | 1 | 2 | Low |
| Suzuki et al., 2016 [82] | 1 | 1 | 0 | 0 | 1 | 0 | 1 | 1 | 5 | Medium |
| Tang et al., 2018 [55] | 1 | 1 | 1 | 1 | 1 | 1 | 1 | 1 | 8 | High |
| Teye et al., 2017 [56] | 1 | 1 | 1 | 1 | 1 | 1 | 1 | 1 | 8 | High |
| Thomsen et al., 2016 [58] | 1 | 1 | 1 | 1 | 1 | 1 | 1 | 1 | 8 | High |
| Thorsteinsdottir et al., 2019 [59] | 0 | 1 | 0 | 0 | 0 | 1 | 0 | 1 | 3 | Low |
| Trzeciak et al., 2017 [60] | 1 | 1 | 1 | 1 | 1 | 1 | 1 | 1 | 8 | High |
| Tyurin et al., 2017 [61] | 1 | 1 | 0 | 0 | 0 | 0 | 0 | 0 | 2 | Low |
| Wan et al., 2017 [62] | 1 | 1 | 1 | 1 | 1 | 1 | 1 | 1 | 8 | High |
| Wen et al., 2019 [63] | 1 | 1 | 1 | 1 | 1 | 1 | 1 | 1 | 8 | High |
| Wong et al., 2018 [88] | 1 | 1 | 0 | 1 | 0 | 1 | 1 | 1 | 6 | Medium |
