# Supplementary figures and images for: Genetics and Epigenetics of Atopic Dermatitis: An Updated Systematic Review
Source: Genes (Basel). 2020 Apr 18;11(4):442. doi: 10.3390/genes11040442 (PMC7231115; doi:10.3390/genes11040442)

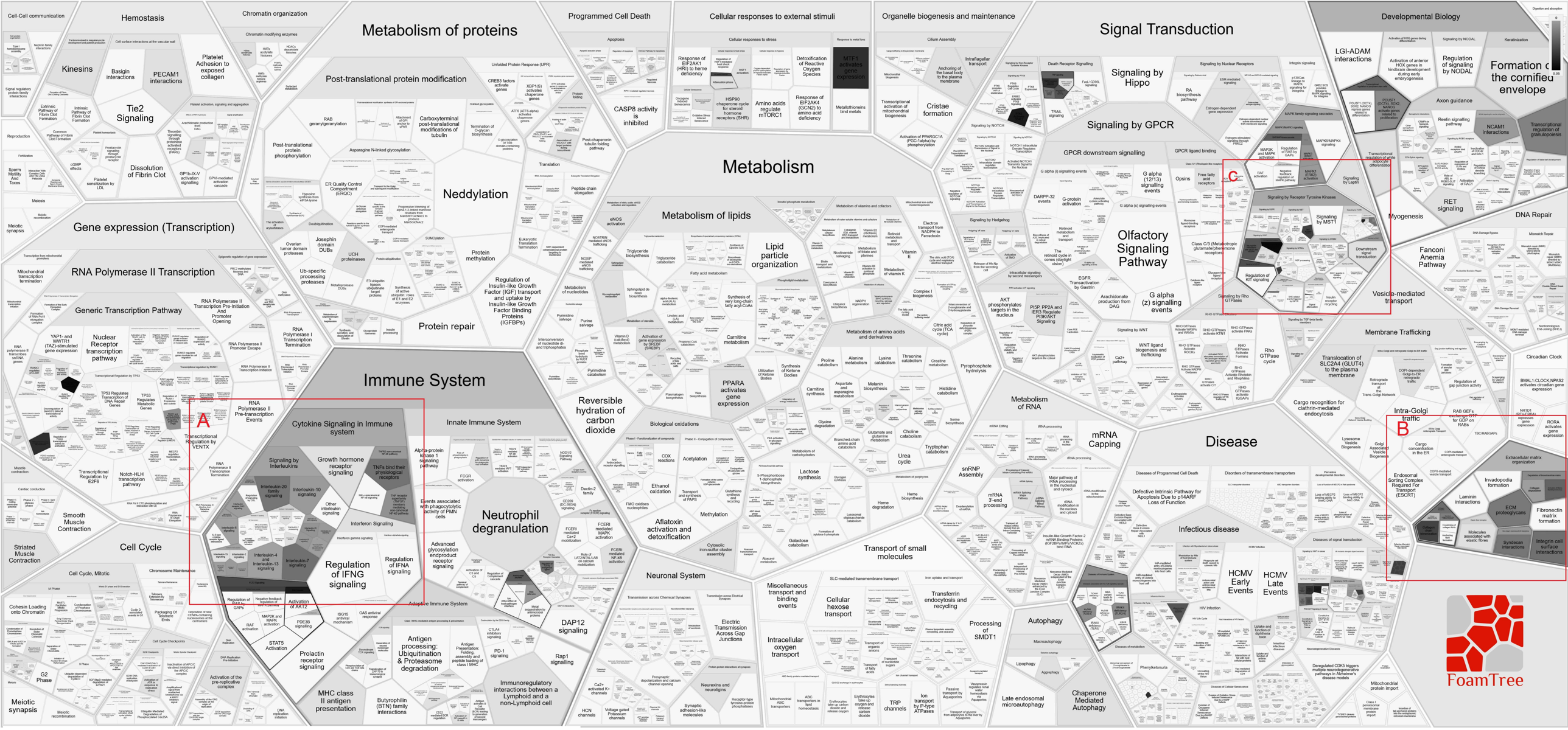

Supplement: Supplementary file 1 [file genes-11-00442-s001.zip › FIGURE S1.pdf]
